# Deciphering spatial heterogeneity by multimodal spatial transcriptomics modelling with SpatialModal

**DOI:** 10.1093/bioinformatics/btag540

**Published:** 2026-07-21

**Authors:** Xingyi Li, Dongmin Zhao, Xiangting Jia, Gaoyuan Du, Jialuo Xu, Yang Qi, Yiqi Chen, Yingfu Wu, Jia Gu, Junnan Zhu, Xuequn Shang

**Affiliations:** School of Computer Science, Northwestern Polytechnical University, Shaanxi, 710129, China; Faculty of Data Science, City University of Macau, Macau, Macau 999078, China; School of Computer Science, Northwestern Polytechnical University, Shaanxi, 710129, China; School of Computer Science, Northwestern Polytechnical University, Shaanxi, 710129, China; School of Computer Science, Northwestern Polytechnical University, Shaanxi, 710129, China; School of Computer Science, Northwestern Polytechnical University, Shaanxi, 710129, China; School of Computer Science, Northwestern Polytechnical University, Shaanxi, 710129, China; School of Computer Science, Northwestern Polytechnical University, Shaanxi, 710129, China; School of Computer Science, Northwestern Polytechnical University, Shaanxi, 710129, China; Faculty of Data Science, City University of Macau, Macau, Macau 999078, China; State Key Laboratory of Multimodal Artificial Intelligence Systems, Institute of Automation, Chinese Academy of Sciences, Beijing, 100190, China; School of Computer Science, Northwestern Polytechnical University, Shaanxi, 710129, China

## Abstract

**Motivation:**

Advances in spatial transcriptomics (ST) technologies have made it possible to jointly acquire gene expression and histological image information while preserving spatial coordinates. This breakthrough presents unprecedented opportunities for the precise dissection of spatial heterogeneity in complex tissues. However, existing computational methods remain limited in their capacity for effective integration and synergistic modelling of multimodal ST data.

**Results:**

We propose SpatialModal, a multimodal graph learning framework that learns robust joint representations by combining a hierarchical representation strategy with a dual-level contrastive learning mechanism. We perform extensive validation of SpatialModal across diverse ST datasets spanning human and mouse tissues. The results demonstrate that SpatialModal effectively reveals intricate brain architectures in humans and mice, dissects tumour microenvironment heterogeneity in breast cancer, delineates Alzheimer’s disease patterns, and characterizes spatiotemporal developmental trajectories within the embryonic heart, underscoring its capability to decipher the spatial heterogeneity of biological tissues. Furthermore, SpatialModal exhibits remarkable versatility and robustness, maintaining superior efficacy even on unimodal datasets devoid of histological images, thereby ensuring its broad applicability across diverse ST platforms.

**Availability and Implementation:**

SpatialModal is implemented in Python and is freely available at https://github.com/xingyili/SpatialModal. The source code used in this study has been archived on Zenodo at DOI: https://doi.org/10.5281/zenodo.21264356. All datasets used in this study are publicly available at https://doi.org/10.5281/zenodo.18220735.

## Graphical Abstract

**Graphical Abstract:**
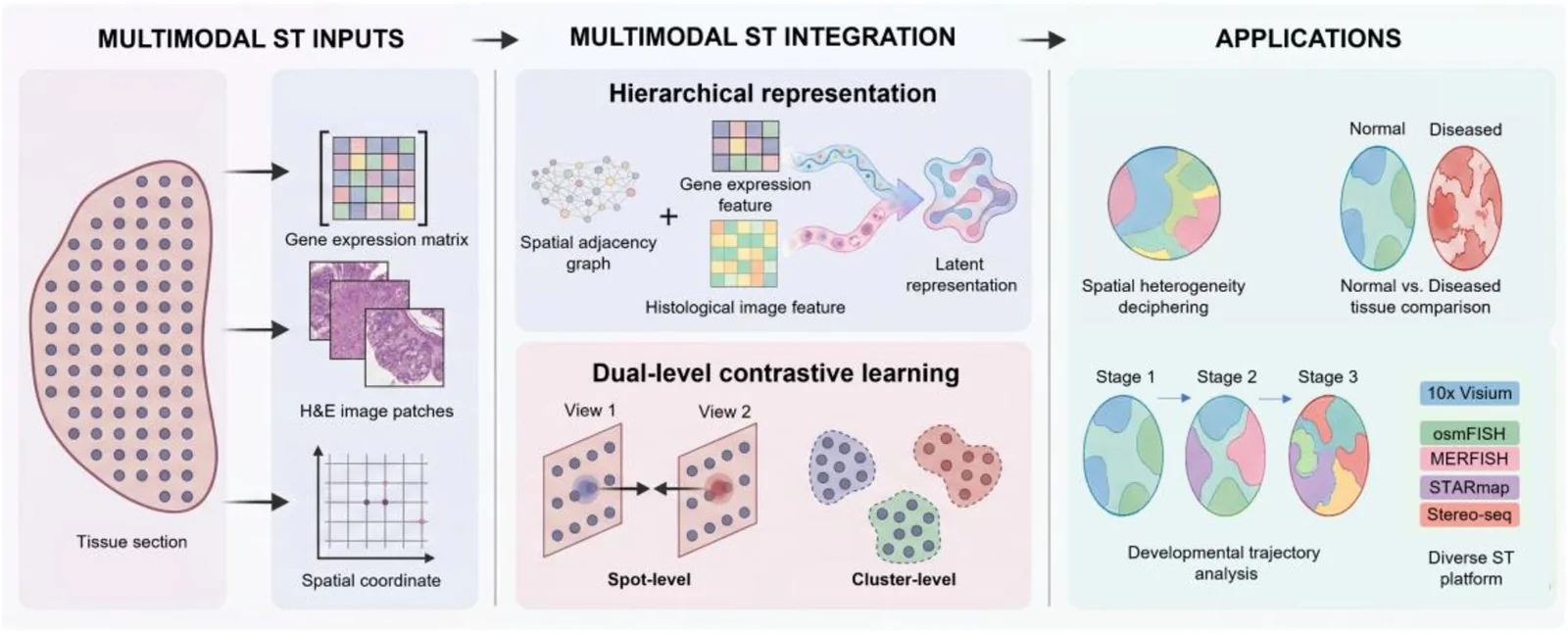

## 1 Introduction

Spatial heterogeneity is a fundamental property of complex tissues, manifested as regional differences in cellular composition, transcriptional programmes, and tissue architecture. Such heterogeneity underlies functional specialization within tissues and plays a central role in diverse biological processes, including development, tissue homeostasis, and disease progression. Therefore, systematically characterizing spatial heterogeneity is essential for understanding how tissue organization relates to biological function. Recent spatial transcriptomics (ST) technologies (Ståhl *et al.* 2016, Moses and Pachter 2022), such as 10× Visium (Ji *et al.* 2020, Rao *et al.* 2020), Stereo-seq (Chen *et al.* 2022a), and multiplexed error-robust fluorescence in situ hybridization (MERFISH) (Chen *et al.* 2015), have created unprecedented opportunities to address this challenge.

An important step toward characterizing spatial heterogeneity in ST data is spatial domain identification, which aims to partition tissue into regions with coherent molecular and structural characteristics. Current spatial domain identification methods can be broadly classified into two categories, namely non-spatial clustering methods and spatial clustering methods. Non-spatial clustering methods, such as K-means, Seurat (Argelaguet *et al.* 2021), and Louvain (Traag *et al.* 2019), rely primarily on gene expression profiles while neglecting spatial context. Consequently, these approaches often struggle to accurately reconstruct continuous tissue structures. To overcome this limitation, spatial clustering methods like STAGATE (Dong and Zhang 2022), GraphST (Long *et al.* 2023), and SpatialGEO (Li *et al.* 2026a) leverage the spatial coordinates to aggregate neighbourhood information using graph neural networks (GNNs) or statistical models. By exploiting spatial proximity, these approaches enforce spatial coherence, facilitating a more precise delineation of tissue microenvironments. In addition, multimodal ST has made it possible to decipher spatial heterogeneity by integrating diverse data modalities within the original tissue sections. Some ST technologies provide matched histological images alongside gene expression profiles and spatial coordinates, most commonly haematoxylin and eosin (H&E) staining. These images provide important morphological information, including cellular morphology, tissue density, and structural boundaries, thereby offering complementary information about tissue architecture and microenvironmental organization. Consequently, the effective integration of multimodal ST data is crucial for accurately deciphering spatial heterogeneity in tissues. Several methods, including SpaGCN (Hu *et al.* 2021), DeepST (Xu *et al.* 2022), stLearn (Pham *et al.* 2023), and STAIG (Yang *et al.* 2025), have attempted to incorporate multiple modalities for spatial heterogeneity analysis. However, in many existing approaches, image features are often treated primarily as auxiliary information to refine spatial graphs, guide denoising, or impose additional regularization. Image features are not deeply integrated with gene features and spatial information to form a joint representation.

To address these challenges, we present SpatialModal, a multimodal graph learning framework that learns robust joint representations by combining a hierarchical representation strategy with a dual-level contrastive learning mechanism. We extensively validate the performance of SpatialModal across diverse ST datasets spanning various tissue types in humans and mice. The results demonstrate that SpatialModal effectively reveals intricate brain structures, tumour microenvironment heterogeneity, Alzheimer’s disease patterns, and spatiotemporal developmental trajectories within the embryonic heart, highlighting its capability to decipher the spatial intricacies of biological tissues. Furthermore, although SpatialModal is designed for multimodal ST data, its architecture can naturally adapt to settings where histological images are unavailable.

## 2 Materials and methods

### 2.1 Overview of SpatialModal

**Figure 1:**
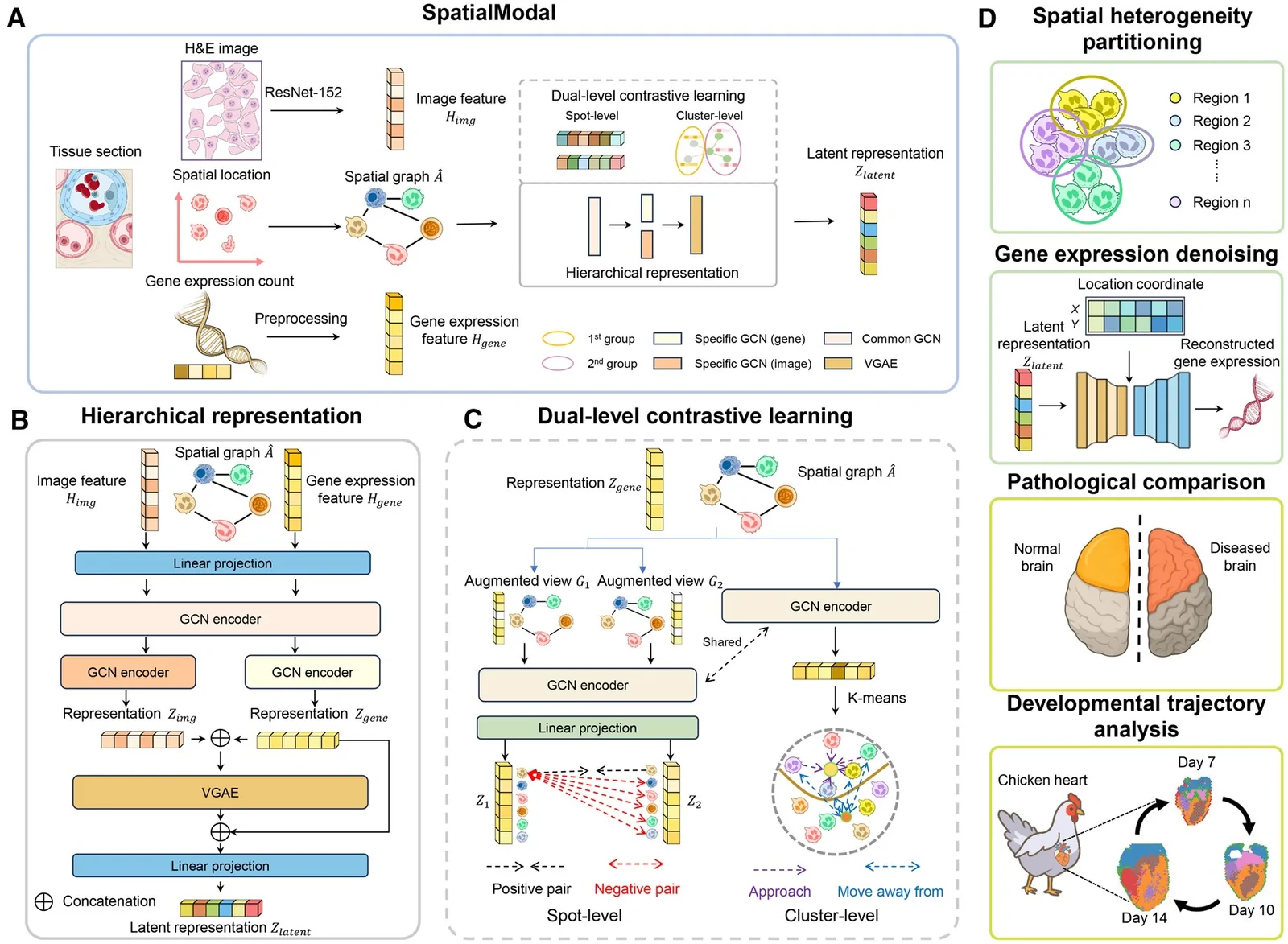
Overview of SpatialModal. (A) SpatialModal is a multimodal graph learning framework that integrates gene expression, spatial coordinates, and histological images through a hierarchical representation module and a dual-level contrastive learning scheme to generate unified latent representations of spots. (B) Hierarchical representation module for multimodal ST integration, in which a common graph convolutional network (GCN) captures shared spatial context, modality-specific GCNs preserve transcriptomic and morphological signals, and a VGAE fuses them into a unified latent space. (C) Dual-level contrastive learning regularizes representation learning at the spot and cluster levels, promoting local consistency and global structural coherence. (D) The learned latent representations support multiple downstream analyses, including spatial heterogeneity partitioning, gene expression denoising, pathological comparison, and developmental trajectory analysis.

SpatialModal is a multimodal graph learning framework that integrates gene expression, spatial coordinates, and histological images to learn unified representations for spatial heterogeneity analysis (Fig. 1a). A hierarchical representation module first encodes these multimodal inputs onto a shared spatial graph (Fig. 1b), followed by a variational graph autoencoder (VGAE) (Kipf and Welling 2016) that integrates the hierarchical features into a unified latent space. To further regularize the representation learning, SpatialModal introduces a dual-level contrastive learning (CL) strategy (Fig. 1c) at the spot and cluster levels, preserving local consistency alongside global tissue organization. The resulting latent representations facilitate the downstream analyses (Fig. 1d).

### 2.2 Data description

We collected publicly available ST datasets from diverse platforms to evaluate SpatialModal across tissues, species, and technologies. These datasets include 12 annotated human dorsolateral prefrontal cortex (DLPFC) sections from three individuals (3498–4789 spots), three mouse brain sections, including sagittal anterior, sagittal posterior, and coronal sections (2695, 3355, and 6050 spots), one human breast cancer section (3798 spots), and two human middle temporal gyrus (MTG) sections from healthy control and Alzheimer’s disease samples (4701 and 4832 spots). We further analysed three chicken heart sections at developmental days 7, 10, and 14 (535, 969, and 1967 spots), as well as mouse somatosensory cortex, hypothalamic, primary visual cortex, and E9.5 embryo datasets containing 4839, 5926, 1020, and 5913 spots, respectively (Table S2).

### 2.3 Data preprocessing

The raw gene expression count matrix is preprocessed using SCANPY (Wolf *et al.* 2018). Genes expressed in fewer than 50 spots or with total counts below 10 are removed. The top 3000 highly variable genes (HVGs) are then selected using the Seurat v3 method (Argelaguet *et al.* 2021), followed by normalization, log transformation, scaling, and clipping at a maximum value of 10. This yields the gene expression matrix $X_{\text{gene}}\in R^{N\times 3000}$, where *N* denotes the number of spots.

For H&E-stained images, local 40×40 pixel patches are cropped from high-resolution tissue images according to spot coordinates, covering the 10× Visium spot area and surrounding morphological context. Each patch is upsampled to 299×299 pixels and fed into a pre-trained ResNet-152 (He *et al.* 2016). The network weights are frozen, and the final fully connected layer is removed to obtain 2048-dimensional image features, forming $X_{\text{img}}\in R^{N\times 2048}$.

Finally, the 3000-dimensional gene expression features and 2048-dimensional image features are projected into a shared latent dimension through two independent linear layers, producing $H_{\text{gene}}\in R^{N\times d_{1}}$ and $H_{\text{img}}\in R^{N\times d_{1}}$, where $d_{1}$ denotes the projected feature dimension.

For datasets without histological images, the image input branch is omitted, and the corresponding image features are replaced by the gene-derived representations.

### 2.4 Spatial graph construction

For each tissue section, we constructed an undirected spatial graph G=(V,E), where each node represents a spot and edges encode spatial proximity between spots. In this study, a unified hybrid k-nearest-neighbour (KNN) and distance-threshold strategy was applied to all datasets for spatial graph construction. Specifically, for each spot, we first calculated the Euclidean distances between this spot and all other spots based on their spatial coordinates, and selected its *k* nearest neighbours. To avoid arbitrary neighbour selection when multiple spots had the same distance as the *k*-th nearest neighbour, we further defined an adaptive distance threshold $d_{i}^{\left(k\right)}$ for each spot *i*, corresponding to the Euclidean distance between spot *i* and its *k*-th nearest neighbour. All spots whose distance to spot *i* was less than or equal to w$d_{i}^{\left(k\right)}$ were also included in its spatial neighbourhood. Therefore, the final spatial graph was constructed as the union of the KNN-based adjacency relationships and the adaptive distance-threshold-based adjacency relationships, and was further converted into an undirected graph. This yielded an adjacency matrix $A\in {\left\{0,1\right\}}^{N\times N}$, where $A_{ij}=1$ indicates an edge between spots *i* and *j*. Self-loops were added by $\tilde{A}=A+I$, followed by symmetric normalization to obtain the model input $\hat{A}={\tilde{D}}^{-\frac{1}{2}}\tilde{A}{\tilde{D}}^{-\frac{1}{2}}$, where $\tilde{D}$ is the degree matrix of $\tilde{A}$.

### 2.5 Hierarchical representation

The hierarchical representation module integrates gene expression and histological image features through shared feature alignment, modality-specific encoding, and VGAE-based fusion. First, $H_{\text{gene}}$ and $H_{\text{img}}$ are projected into a shared feature space using a shared linear transformation. Motivated by the spatial correspondence between matched histological morphology and gene expression in ST data, a predefined proportion of gene-derived features is randomly replaced with the corresponding image-derived features, yielding a perturbed gene representation ${\tilde{H}}_{\text{gene}}$. This cross-modal perturbation is designed to regularize multimodal fusion by encouraging the model to recover transcriptomic signals from morphology-informed and spatial-contextual cues, thereby reducing over-reliance on a single modality. Detailed ablation analyses are provided in Supplementary Note S5.

The perturbed gene features and image features are then encoded by a shared-parameter GCN to capture common spatial patterns across modalities. Subsequently, two modality-specific GCNs are applied to preserve transcriptomic and morphological information, producing the gene representation $Z_{\text{gene}}$ and image representation $Z_{\text{img}}$, respectively. All GCN encoders use the normalized spatial adjacency matrix $\hat{A}$ with learnable linear transformations and nonlinear activations.

The gene representation $Z_{\text{gene}}$ is regularized by the dual-level contrastive learning module and is also concatenated with $Z_{\text{img}}$ to form a joint multimodal representation:


(1) $$Z_{\text{joint}}=[Z_{\text{img}}||Z_{\text{gene}}]\in R^{N\times d_{2}}.$$

The joint representation is further fed into a VGAE to learn a probabilistic multimodal fusion representation. The VGAE encoder estimates the mean μ and standard deviation σ, and the fused representation is sampled through reparameterization:


(2) Z=μ+∈⊙σ, ∈∼N(0,I),

where ⊙ denotes element-wise multiplication. Finally, *Z* is concatenated with $Z_{\text{gene}}$ and projected to obtain the final latent representation:


(3) $$Z_{\text{latent}}=\text{ELU}([Z_{\text{gene}}||Z]W_{f}+b_{f}).$$

where ELU denotes the exponential linear unit activation function.

### 2.6 Dual-level contrastive learning

*Spot-level contrastive learning:* At the spot level, two augmented graph views $G_{1}=({\hat{Z}}_{\text{gene},1},{\hat{A}}_{1})$ and $G_{2}=({\hat{Z}}_{\text{gene},2},{\hat{A}}_{2})$ are generated from $Z_{\text{gene}}$ and $\hat{A}$ through random edge dropping and feature masking. A shared graph encoder and a two-layer nonlinear projection head are then applied to obtain projection vectors $h_{1}$ and $h_{2}$. The spot-level objective is optimized using a symmetric InfoNCE loss:


(4) $$L_{\text{spot}}=\frac{1}{2}(\ell (h_{1},h_{2})+\ell (h_{2},h_{1})),$$

where ℓ(·) denotes the temperature-scaled InfoNCE loss based on cosine similarity after $L_{2}$ normalization, with the temperature coefficient τ set to 0.4. This objective encourages the same spot under different perturbations to have similar representations while separating it from other spots.

*Cluster-level contrastive learning:* At the cluster level, the same shared encoder is applied to $Z_{\text{gene}}$ to obtain cluster-level representations $Z_{\text{clu}}$. K-means is then performed on $Z_{\text{clu}}$ to assign pseudo-labels and compute cluster prototypes. The cluster-level contrastive loss is defined as:


(5) $$L_{\text{cluster}}=-\frac{1}{N}\sum_{i=1}^{N}\;\text{log}\;\frac{\;\text{exp}(\text{cos}(z_{\text{clu},i},c_{y_{i}})/\tau )}{\sum_{k=1}^{K}\;\text{exp}\;(\text{cos}(z_{\text{clu},i},c_{k})/\tau )},$$

where $c_{k}$ is the prototype of cluster *k*, and $y_{i}$ is the pseudo-label of spot *i*. This loss encourages spots with similar biological properties to aggregate around their assigned prototypes in the latent space.

### 2.7 Model training of SpatialModal

SpatialModal is trained by jointly optimizing reconstruction, VGAE, and contrastive objectives.

*Reconstruction loss.* The reconstruction loss preserves gene expression semantics by reconstructing the original HVG feature space from both the final latent representation $Z_{\text{latent}}$ and the gene representation $Z_{\text{gene}}$ for perturbed spots. Specifically, the global reconstruction term $L_{\text{global}}$ is computed from all spots, while the masked reconstruction term $L_{\text{mask}}$ is computed on the subset M whose gene features are replaced by image features. The reconstruction loss is:


(6) $$L_{\text{rec}}=\lambda_{1}L_{\text{global}}+\lambda_{2}L_{\text{mask}}.$$

*VGAE loss.* The VGAE loss preserves spatial topology and regularizes the latent distribution. It consists of a graph reconstruction term $L_{\text{graph}}$, which reconstructs the augmented adjacency matrix $\tilde{A}$ from the fusion representation *Z*, and a Kullback–Leibler (KL) divergence term $L_{\text{KLD}}$, which aligns the approximate posterior with a standard normal prior:


(7) $$L_{\text{vgae}}=\lambda_{3}L_{\text{graph}}+\lambda_{4}L_{\text{KLD}}.$$

*Contrastive loss.* The contrastive objective combines the spot-level and cluster-level contrastive losses:


(8) $$L_{\text{con}}=\lambda_{5}L_{\text{spot}}+\lambda_{6}L_{\text{cluster}}.$$

Finally, the total training objective is:


(9) $$L_{\text{total}}=L_{\text{rec}}+L_{\text{vgae}}+L_{\text{con}},$$

where $\lambda_{1}$–$\lambda_{6}$ are balancing hyperparameters.

The model architecture and training hyperparameters of SpatialModal are summarized in Table S3.

## 3 Results

### 3.1 SpatialModal accurately resolves cortical layers in human brain

To evaluate the performance of SpatialModal in spatial domain detection, we first apply it to the human DLPFC (Maynard *et al.* 2021) dataset generated by the 10x Visium platform. This dataset comprises 12 tissue sections from three adult donors, spanning six cortical layers (Layers 1–6) and the white matter (WM), with manual annotations serving as the ground truth. We benchmark SpatialModal against several state-of-the-art methods (Table S1) using the Adjusted Rand Index (ARI) and Normalized Mutual Information (NMI) (Supplementary Note S1).

**Figure 2:**
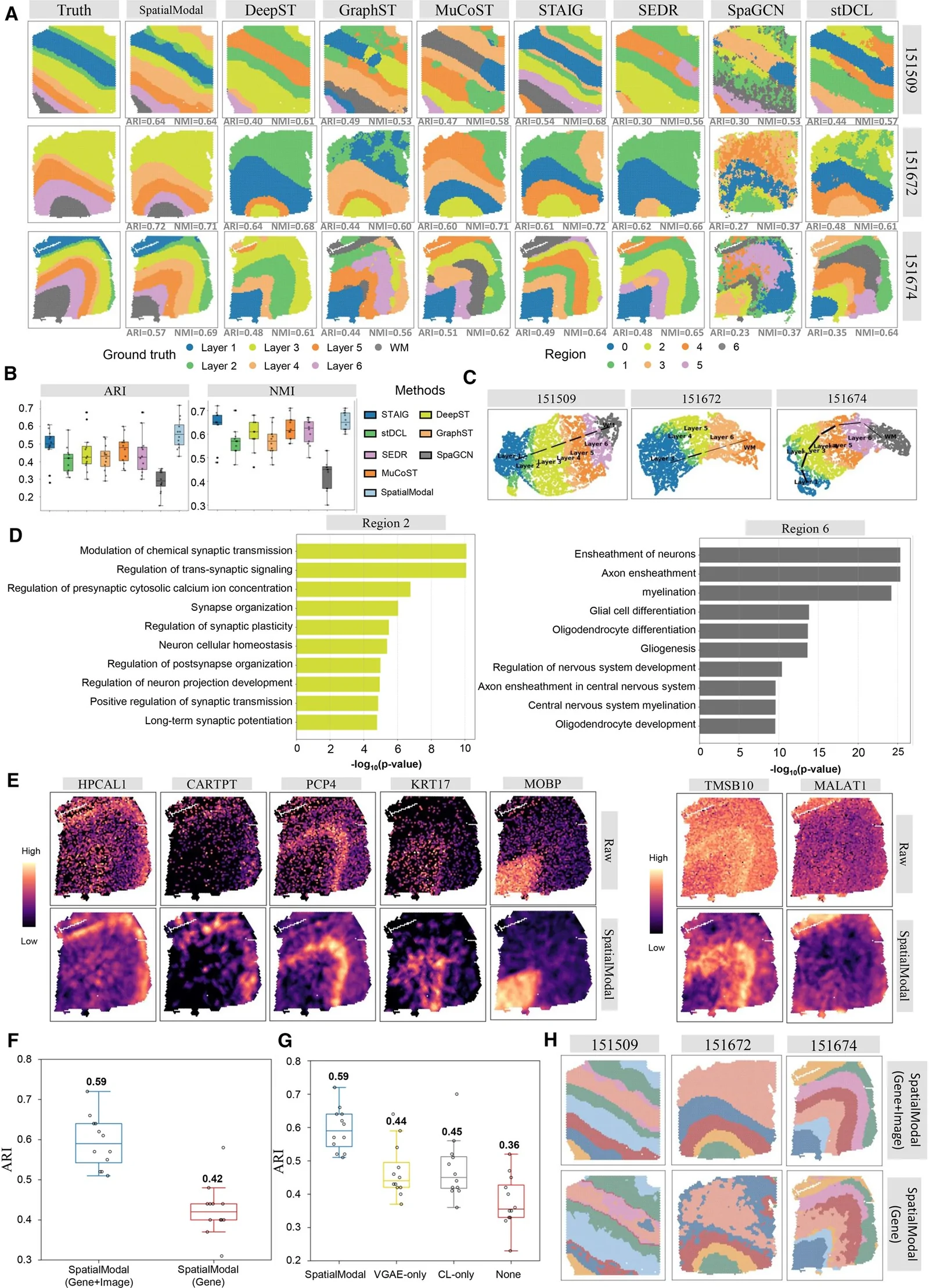
SpatialModal accurately resolves cortical layers in human DLPFC. (A) Annotations and clustering results on three DLPFC tissue sections (151 509, 151 672, 151 674), with corresponding ARI and NMI values. (B) Boxplots showing the ARI and NMI values for eight methods across 12 DLPFC tissue sections. (C) UMAP and PAGA visualization of latent representations learned by SpatialModal on the three tissue sections. The UMAP plots are coloured by ground truth annotations. (D) GO enrichment analysis for region 2 and region 6 identified by SpatialModal, showing the top ten significantly enriched terms. (E) Raw and denoised gene expression patterns of known marker genes, including *HPCAL1* (Region 1), *CARTPT* (Region 2), *PCP4* (Region 3), *KRT17* (Region 4), and *MOBP* (Regions 5/6), as well as newly identified marker genes, including *TMSB10* (Regions 3/4) and *MALAT1* (Region 0). (F) Boxplots of ARI scores across 12 DLPFC sections for SpatialModal under multimodal and gene-only input settings. (G) Boxplots of ARI scores across 12 DLPFC sections for the full SpatialModal model and three ablated settings (VGAE-only, CL-only, and None). (H) Representative spatial domain maps for three DLPFC sections under gene-only and multimodal input settings.

The results show that SpatialModal achieves an average ARI of 0.59 and an average NMI of 0.65 across the 12 tissue sections. This performance substantially outperforms other methods (Figs. 2a and b, S1). This performance gain is stable across most sections and is particularly prominent in specific tissue sections (e.g. 151 670, 151 509, and 151 672), with ARI increasing by up to 100% and NMI by approximately 30%. On representative tissue sections from the three different donors (151 509, 151 672, and 151 674), SpatialModal clearly captures the layered structures that match the manual annotations. Furthermore, Uniform Manifold Approximation and Projection (UMAP) (Healy and McInnes 2024) visualization reveals a clear cortical hierarchy among the six layers and WM. Partition-based Graph Abstraction (PAGA) (Wolf *et al.* 2019) further reveals the topological relationships among these cortical layers (Figs. 2c and S2).

To validate the biological relevance of the SpatialModal-identified layers, we first perform differential gene expression (DEG) analysis for each layer. We find that the identified layers are significantly enriched with known marker genes. Subsequent Gene Ontology (GO) enrichment analysis reveals that the DEGs are significantly enriched in critical biological processes, including synaptic transmission and plasticity, nervous system development, and cellular homeostasis. These findings underscore the intricate molecular mechanisms that underpin the advanced cognitive functions of the prefrontal cortex (Fig. 2d and Supplementary Figs. S3, S4, and S5).

Moreover, we conduct a gene expression denoising analysis (Supplementary Note S4). The results demonstrate that SpatialModal significantly enhances the expression patterns of marker genes and improves their spatial continuity (Figs. 2e and S6). Notably, the expression profiles of certain genes after denoising can more clearly reveal the layer-specific patterns that are often obscured by noise in the raw data. For instance, the expression of the *TMSB10* (Xiong *et al.* 2022) gene is highly enriched in Layer 5 (region 3) after denoising. Its encoded protein is closely associated with actin cytoskeleton remodelling and synaptic plasticity processes occurring within layer 5. Similarly, the expression of the *MALAT1* (Tripathi *et al.* 2010) gene in Layer 1 (region 0) after denoising is clearly distinguished. As a regulator of gene expression, MALAT1 potentially plays a role in maintaining the molecular homeostasis required for the unique synaptic microenvironment and signal processing characteristics of layer 1.

To assess the contribution of histological images to deciphering spatial heterogeneity, we systematically compare the performance of SpatialModal on 12 DLPFC tissue sections using both multimodal and gene-only inputs. The results reveal that integrating histological images robustly enhances spatial domain identification across all 12 DLPFC tissue sections (Fig. 2f), effectively eliminating local noise and regional fragmentation to facilitate a highly precise dissection of the fine-grained cortical laminar architecture (Fig. 2h).

To delineate the specific contributions of the dual-level contrastive learning module and the VGAE fusion module, we conduct ablation studies across the 12 DLPFC tissue sections. Compared with the full SpatialModal model (median ARI = 0.59), the removal of either or both components precipitates a marked decline in performance (Fig. 2g). Notably, replacing both modules with a linear layer (None) yields the lowest median ARI (0.36), while retaining only the VGAE fusion module (VGAE-only) or the dual-level contrastive learning module (CL-only) achieves median ARIs of 0.44 and 0.45, respectively. These results highlight that while each individual module confers a partial performance gain, their concurrent integration is required. Together, they function synergistically to maximize both the accuracy and structural robustness of spatial domain identification.

### 3.2 SpatialModal delineates complex mouse brain structures

To further evaluate the analytical performance of SpatialModal across complex tissue architectures, we apply the algorithm to mouse brain 10× Visium datasets, which comprise two consecutive sagittal sections (anterior and posterior) and one coronal section.

**Figure 3:**
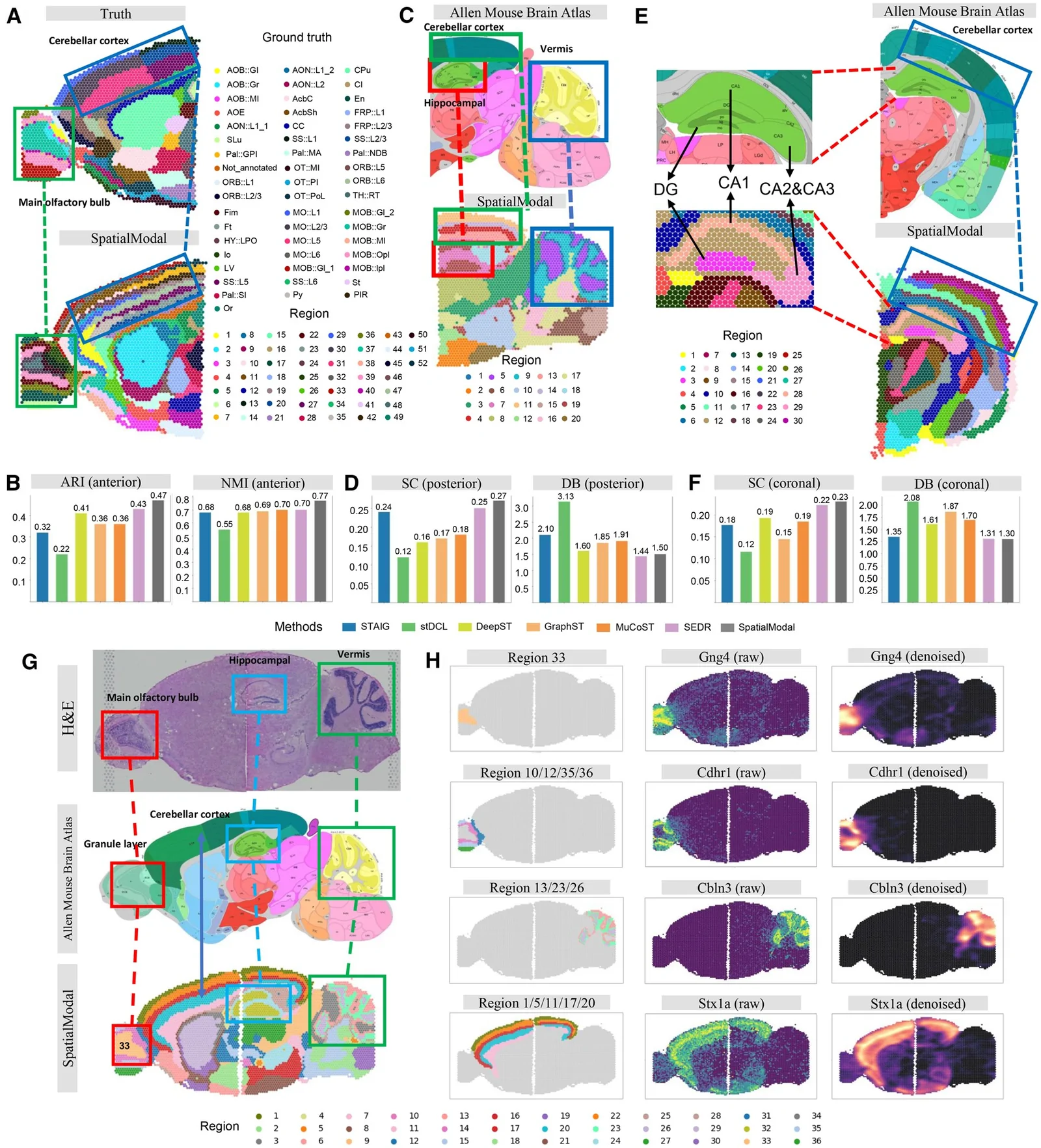
SpatialModal delineates complex mouse brain structures. (A) Annotations and SpatialModal clustering results for the sagittal anterior mouse brain section. (B) Barplots of ARI and NMI values for seven methods on the sagittal anterior mouse brain section. (C) Allen Mouse Brain Atlas and SpatialModal clustering results for the sagittal posterior mouse brain section. (D) Barplots of SC and DB values for seven methods on the sagittal posterior mouse brain section. (E) Allen Mouse Brain Atlas and SpatialModal clustering results for the coronal mouse brain section. (F) Barplots of SC and DB values for seven methods on the coronal mouse brain section. (G) H&E images, Allen Mouse Brain Atlas, and SpatialModal clustering results for the jointly analysed sagittal anterior and posterior mouse brain sections. (H) Raw and denoised expression patterns of representative marker genes for SpatialModal-identified regions.

On the sagittal anterior section with ground truth annotations, SpatialModal demonstrates superior performance, with quantitative metrics substantially outperforming baseline methods (ARI = 0.47, NMI = 0.77), and achieves precise identification of the cerebellar cortex and the main olfactory bulb (MOB) (Figs. 3a and b, S7a). For the unannotated posterior and coronal sections, we employ the Silhouette Coefficient (SC) and Davies–Bouldin (DB) index for unsupervised evaluation (Supplementary Note S1). The results indicate that SpatialModal maintains superior clustering performance across both datasets (Figs. 3d and f, S7b and c). Specifically, in the sagittal posterior section, SpatialModal not only resolves the cerebellar cortex into four distinct sub-layers but also accurately identifies key anatomical regions, including the hippocampus and the vermis, yielding results highly consistent with the Allen Mouse Brain Atlas (Fig. 3c). Similarly, in the coronal section, SpatialModal successfully resolves the cerebellar cortex, the dentate gyrus (DG), and the Cornu Ammonis (CA), further enabling fine-grained characterization of CA sub-regions (CA1, CA2, and CA3) (Fig. 3e).

We further conduct joint modelling of the sagittal anterior and posterior sections (Supplementary Note S3), corroborating our findings with the Allen Mouse Brain Atlas (Fig. 3g). The results confirm that SpatialModal achieves precise structural identification and anatomical alignment across disparate sections. Within the MOB, SpatialModal-identified region 33 shows strong concordance with the granule layer of the Allen Mouse Brain Atlas and the hyperchromatic, cell-dense regions in H&E staining. Meanwhile, SpatialModal also accurately identifies the curved contours of the vermis (Fig. S7d). Furthermore, SpatialModal demonstrates robustness at anatomical junctions by resolving the cerebellar cortex into six distinct cortical layers and accurately delineating the morphological boundaries of the hippocampus, thereby highlighting its consistency in cross-section structural recognition. Subsequently, we evaluate SpatialModal’s capacity for gene expression denoising across sections (Fig. 3h). Representative marker genes, such as *Gng4* and *Cdhr1* in the MOB and *Cbln3* in the vermis, show significantly enhanced patterns following gene expression denoising by SpatialModal. Notably, for *Stx1a*, a gene exhibiting sparse and poorly defined laminar patterns in the raw data, SpatialModal effectively recovers biologically meaningful signals and yields a denoised expression profile that faithfully reflects the hierarchical spatial organization of the cortical anatomy.

### 3.3 SpatialModal reveals the tumour microenvironment of breast cancer

**Figure 4:**
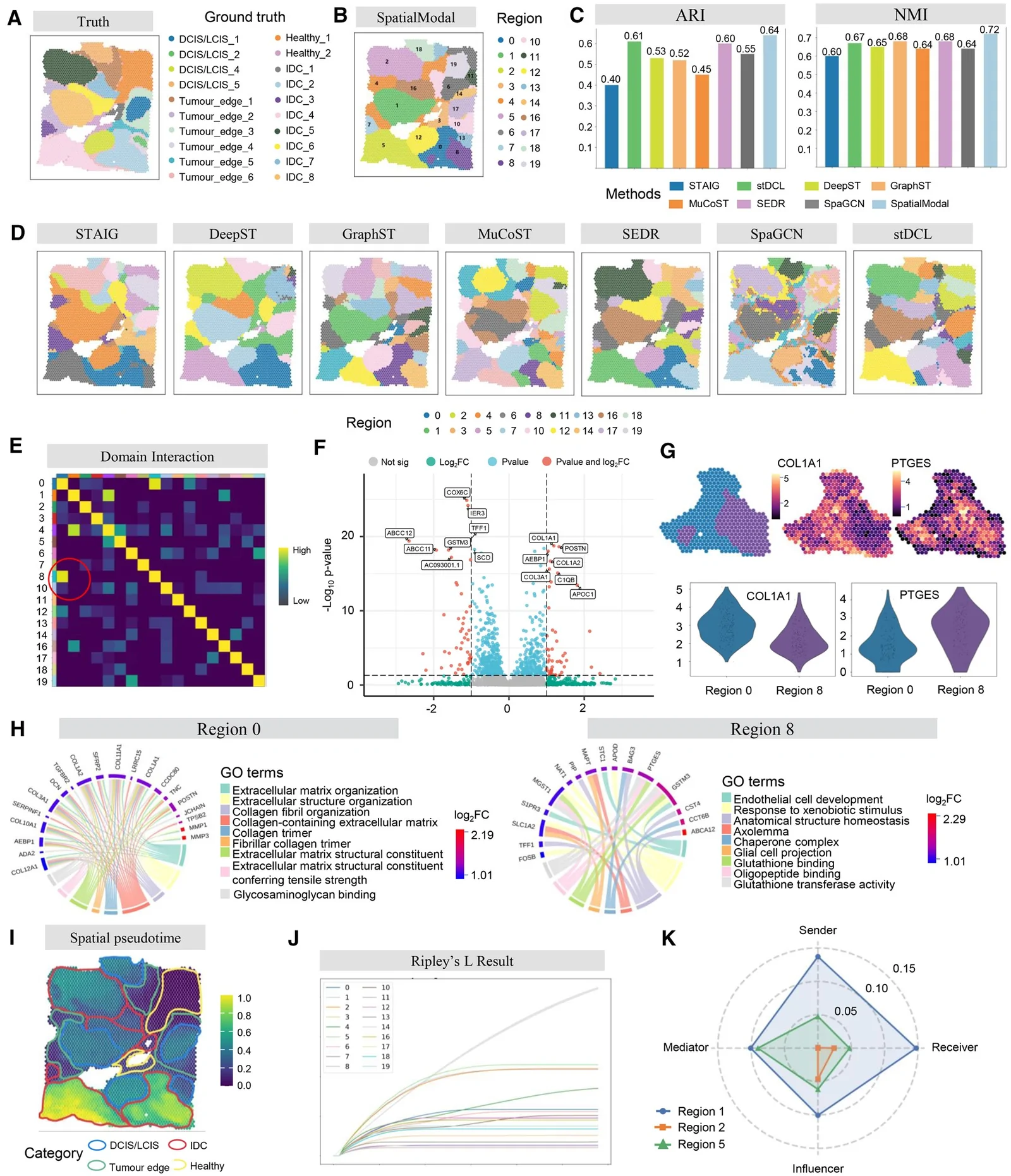
SpatialModal reveals the tumour microenvironment of breast cancer. (A) Annotations and SpatialModal clustering results for the breast cancer tissue section. (B) Spatial domains identified by SpatialModal on the human breast cancer dataset. (C) Clustering performance metrics (ARI and NMI) of SpatialModal compared with seven other methods. (D) Spatial domains identified by seven competing methods on the human breast cancer dataset. (E) Heatmap illustrating the degree of spatial interaction between domains identified by SpatialModal. (F) Volcano plot of DEGs between region 0 and region 8. Red dots on the right represent genes upregulated in region 0, while red dots on the left represent genes upregulated in region 8. (G) Expression patterns of COL1A1 and PTGES genes, with regional annotations for region 0 and region 8 (top) and corresponding violin plots (bottom). (H) GO enrichment analysis of the DEGs between region 0 and region 8. (I) Spatial pseudotime inference by SpatialModal. Higher values indicate regions that appear at later stages of progression. (J) Spatial clustering analysis using Ripley’s L-function. Curves represent the degree of spatial aggregation for each identified domain across varying radii *r*. (K) Radar chart delineates the influence exerted by regions 1, 2, and 5 acting as senders, receivers, mediators, and influencers within the cell-cell communication network.

To validate the performance of SpatialModal in revealing the tumour microenvironment, we apply SpatialModal to a 10× Visium human breast cancer dataset. This dataset comprises four distinct regional categories: ductal/lobular carcinoma in situ (DCIS/LCIS), invasive ductal carcinoma (IDC), tumour edge, and healthy tissue (Fig. 4a). The results show that the spatial domains identified by SpatialModal exhibit the highest consistency with the annotations (ARI = 0.64, NMI = 0.72, Fig. 4b–d). Furthermore, SpatialModal identifies spatial domains corresponding to three typical DCIS/LCIS subtypes (DCIS/LCIS_1, DCIS/LCIS_4, DCIS/LCIS_5) and four typical IDC subtypes (IDC_3, IDC_4, IDC_5, IDC_7).

Importantly, SpatialModal reveals significant intra-regional heterogeneity within the original IDC_2 area, resolving it into two functionally distinct subregions (region 0 and region 8). This finding is also consistent with results from GraphST, SpaGCN, and SEDR (Xu *et al.* 2024), and the domain interaction analysis confirms a strong association between these two regions (Fig. 4e). Subsequent DEGs and GO enrichment analyses highlight the functional specificity of these two subregions (Fig. 4f–h).

In region 0, genes such as *COL1A1*, *C1QB*, and *APOC1* are significantly upregulated. Notably, *COL1A1* typically indicates tumour stroma hardening, while *C1QB* and *APOC1* serve as markers for tumour-associated macrophages. These upregulated genes are involved in GO enrichment terms such as tissue remodelling, fibrosis, and structural support. Conversely, genes including *PTGES* and *IER3* are significantly upregulated in region 8, where *PTGES* catalyses the production of immunosuppressive factors in the tumour microenvironment and *IER3* regulates apoptosis and inflammatory signalling pathways. The GO enrichment analysis results show that region 8 is primarily enriched in terms related to metabolism and cellular homeostasis. This suggests that distinct immunological differences exist even between adjacent tumour domains within IDC_2, potentially leading to differential responses to immunotherapy.

To explore the spatial continuity and potential evolutionary dynamics of breast cancer, we utilize SpatialModal to construct a spatial pseudotime map (Fig. 4i). The analysis suggests a spatially organized progression-like continuum from healthy tissue through the tumour edge and carcinoma in situ to invasive ductal carcinoma. This alignment with known biological findings underscores SpatialModal’s capacity to characterize complex tissue evolution (Lips *et al.* 2022, Wang *et al.* 2024). Furthermore, Ripley’s L-function analysis validates that three specific spatial domains—region 1 (DCIS/LCIS_5), region 2 (IDC_5), and region 5 (IDC_4)—possess significant spatial clustering characteristics (Fig. 4j). Considering the pivotal role of the C-X-C motif chemokine ligand (CXCL) signalling pathway (Steele *et al.* 2016) in the breast cancer microenvironment and immune regulation, we focus on comparing communication differences among these three regions within the CXCL signalling pathway (Fig. 4k). The results show that region 1 serves as a central hub within the CXCL communication network, showing stronger communication activity as both a signal sender and receiver compared to region 2 and region 5. This finding suggests that carcinoma in situ may actively drive tumour microenvironment remodelling by mediating more robust intercellular communication.

### 3.4 SpatialModal reveals spatial patterns in Alzheimer’s disease

**Figure 5:**
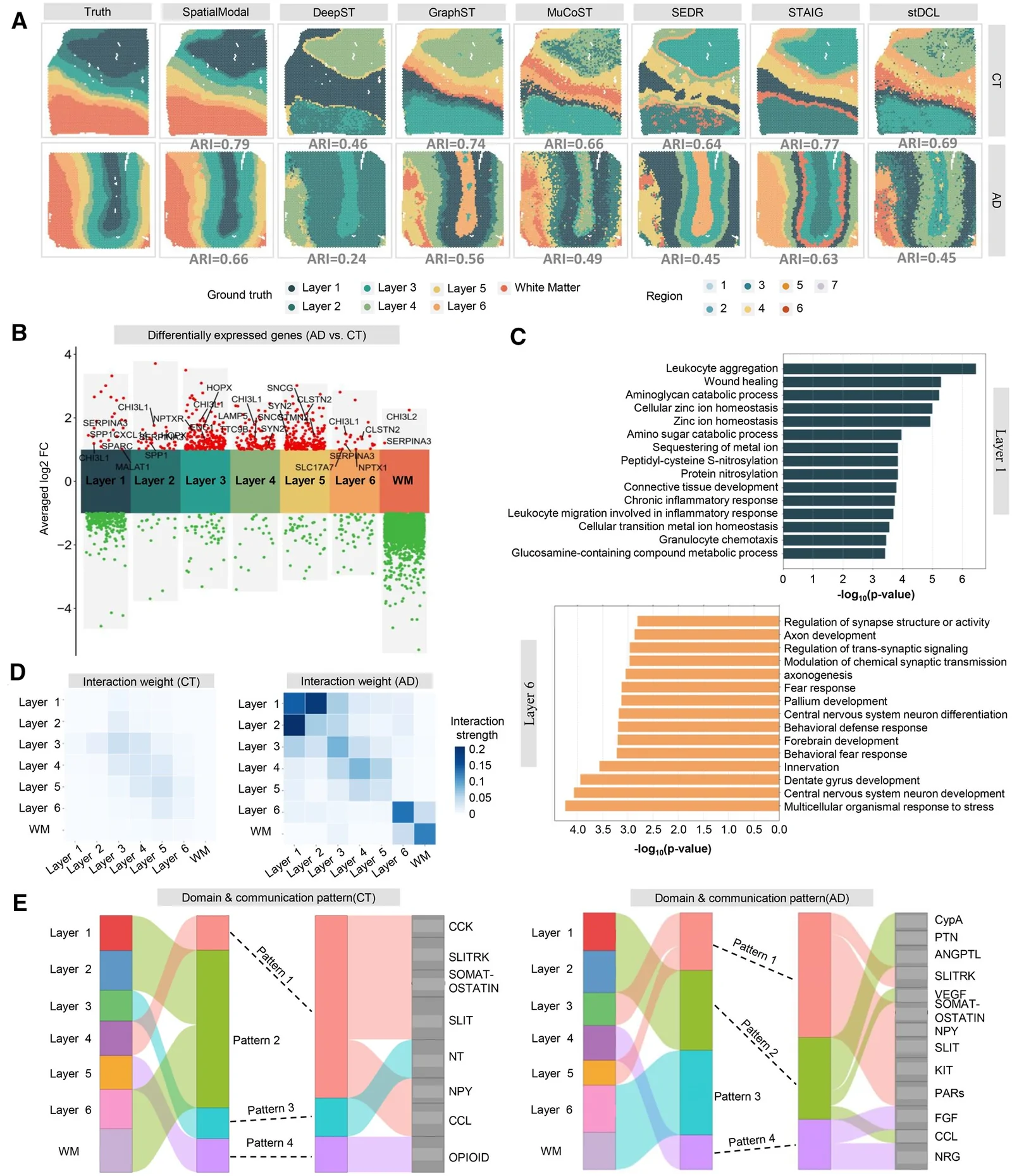
SpatialModal reveals spatial patterns in Alzheimer’s disease. (A) Annotations and spatial clustering results for SpatialModal and six comparative methods on the CT and AD tissue sections, with corresponding ARI values. (B) DEG analysis (AD vs. CT). Red dots indicate upregulated genes and green dots indicate downregulated genes. (C) GO enrichment analysis for upregulated DEGs in Layer 1 and Layer 6 of the AD tissue section, showing the top 15 significantly enriched GO terms. (D) Heatmaps illustrate the interaction weights between SpatialModal-identified spatial domains in the CT (left) and AD (right) tissue sections. (E) Sankey diagrams mapping the dominant signalling pathways, including cholecystokinin (CCK) and cyclophilin A (CypA), and their corresponding communication patterns (Patterns 1–4) to each spatial domain for both CT (left) and AD (right) tissue sections.

To investigate spatial heterogeneity in Alzheimer’s disease, we employ SpatialModal on the 10x Visium human MTG dataset (Chen *et al.* 2022b). We analysed two MTG tissue sections, including one section from an individual with Alzheimer’s disease (AD) and one section from a healthy control (CT). It features annotations for seven spatial domains (six cortical layers and one WM layer). SpatialModal demonstrates robust spatial domain identification capabilities, with ARI scores of 0.79 (CT) and 0.66 (AD) (Fig. 5a). It accurately captures the cortical laminar organization with high ground truth concordance. Particularly in challenging regions such as cortical borders and WM, SpatialModal displays clear, spatially coherent layers, whereas benchmarking methods often show less clear architectural resolution.

To elucidate layer-specific molecular mechanisms of AD, we calculate DEGs comparing AD and CT tissue sections across each cortical layer (Fig. 5b). We then conduct GO enrichment analysis on upregulated genes in AD (Figs. 5c and S8). Results reveal that AD pathology involves widespread molecular dysregulation, ranging from the disruption of immune homeostasis in superficial layers to neuronal functional impairment in deep layers, thereby reflecting the complexity of the cortical pathological microenvironment. Notably, although the majority of genes in the WM are downregulated, the upregulation of *SERPINA3* (Jeong *et al.* 2025) and *CHI3L2* (Moreno-Rodriguez *et al.* 2020) underscores the prominence of neuroinflammation in WM pathology.

We further explore the impact of AD on cell communication by characterizing the changes in communication intensity across cortical layers (Fig. 5d). In the CT tissue section, the cell communication network is relatively sparse and primarily concentrated within Layers 3–5. In contrast, the AD tissue section not only exhibits significantly enhanced self-loop communication across multiple layers but also establishes aberrantly active cross-layer interaction networks, such as between Layer 1 and Layer 2, and between Layer 6 and the WM. These findings indicate that AD induces the dysregulation of cortical communication.

Finally, a comparison of cell communication patterns between the two tissue sections reveals a widespread remodelling of communication landscapes in the MTG associated with AD (Fig. 5e). Specifically, the CT tissue section is dominated by homeostatic signals that maintain neuronal connectivity [e.g. SLIT (Proenca *et al.* 2011)] and the excitation/inhibition balance [e.g. somatostatin (Song *et al.* 2021) and neuropeptide Y (NPY) (Zalocusky *et al.* 2021)]. Conversely, the contribution of these homeostatic signals decreases in the AD tissue section, which is instead overtaken by pathological signalling pathways driven by neuroinflammation [e.g. pleiotrophin (PTN) and cyclophilin A (CypA)] (Kim *et al.* 2025, Newton *et al.* 2025), angiogenesis [vascular endothelial growth factor (VEGF) (Lee *et al.* 2025)], and injury response [KIT (Dubois *et al.* 2023)].

### 3.5 SpatialModal reveals spatiotemporal developmental trajectories in the embryonic heart

**Figure 6:**
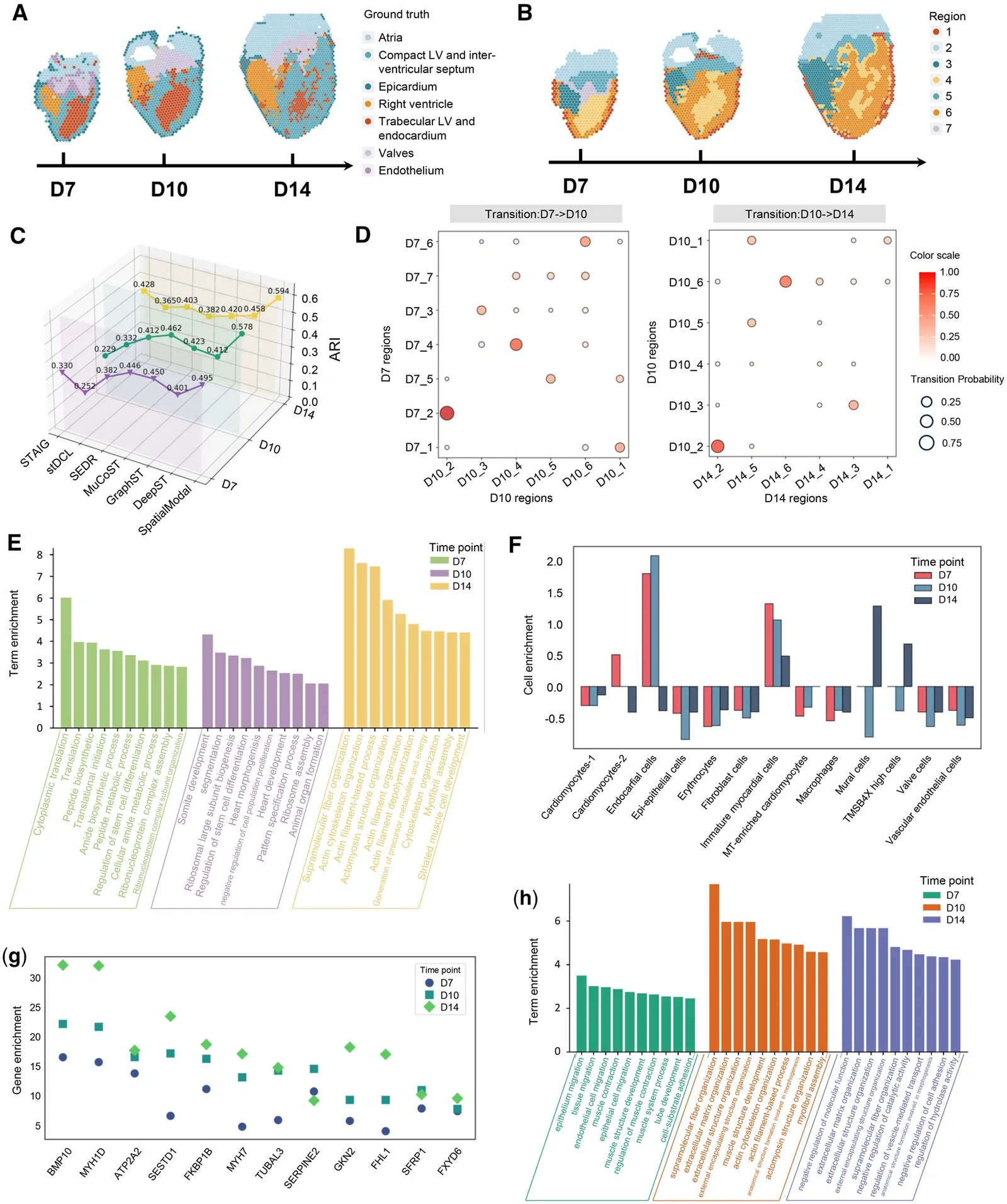
SpatialModal reveals spatiotemporal developmental trajectories in the embryonic heart. (A) Annotations of the chicken heart tissue sections at three developmental stages (D7, D10, D14). (B) Spatial domains identified by SpatialModal on the three time-series tissue sections. (C) 3D line plot comparing clustering accuracy (ARI) for SpatialModal and six comparative methods across the three time-series tissue sections. (D) Dotplots of the optimal transport (OT) transition probabilities between domains across the three developmental stages. (E) GO enrichment of the trabecular LV and endocardium across the three developmental stages. (F) Cell enrichment of various cell types within the trabecular LV and endocardium regions across the three developmental stages. (G) Gene enrichment of selected marker genes within the atrial region across three developmental stages. (H) GO enrichment of the atrial regions across three developmental stages.

To further validate the efficacy of SpatialModal in deciphering spatial heterogeneity using spatiotemporal transcriptomics data, we utilize a chicken heart development dataset from the 10x Visium platform, spanning three developmental stages: day 7 (D7), day 10 (D10), and day 14 (D14) (Mantri *et al.* 2021). This dataset contains annotations for seven spatial domains as well as cell types (Figs. 6a and S9). In the spatial domain identification task, SpatialModal’s results are highly consistent with manual annotations, accurately delineating key structures such as the trabecular left ventricle (LV) and endocardium, atria, and right ventricle (Fig. 6b). Compared with benchmarking methods, SpatialModal achieves the highest ARI across all three developmental stages (Figs. 6c and S10).

Subsequently, we employ the Waddington optimal transport (WOT) (Schiebinger *et al.* 2019) algorithm to evaluate the capacity of SpatialModal to capture regional dynamic evolution during cardiac development (Fig. 6d). The results demonstrate that SpatialModal successfully identifies the expected temporal evolution patterns of the trabecular LV and endocardium (region 4). Specifically, the transfer probability for this region reaches 0.529 between D7 and D10, but significantly decreases to 0.075 between D10 and D14, indicating a dramatic state transition. To elucidate the underlying mechanisms of this transition, we perform GO enrichment analysis and cell composition analysis (Fig. 6e and f). During the D7–D10 stage, the trabecular LV and endocardium are predominantly composed of endocardial cells and immature cardiomyocytes, with functions primarily enriched in protein synthesis and morphogenesis, exhibiting strong developmental continuity. By D14, as endocardial cell populations decline and mural cells become significantly enriched, the functional focus shifts toward myofibril assembly, striated muscle development, and energy metabolism. This transition from morphological construction to mature pumping function is consistent with the conclusion of the original dataset publication that the trabecular ventricle gradually disappears during late development (Mantri *et al.* 2021).

Unlike the trabecular LV and endocardium, SpatialModal reveals high temporal continuity within the atrial region throughout development, with transfer probabilities maintained at high levels of 0.726 (D7–D10) and 0.605 (D10–D14). We subsequently perform DEG analysis to examine the transcriptomic signatures of the atria at different stages (Fig. 6g), identifying significant enrichment of *BMP10* and *MYH1D* across all stages. *BMP10* is a recognized atrial marker essential for maintaining the atrial cardiomyocyte phenotype, while *MYH1D* serves as a marker for identifying cells of atrial origin. Further GO enrichment analysis reveals that terms related to muscle structural development persist through D7 and D10, marking the initial establishment of atrial contractile function. Conversely, terms associated with extracellular matrix organization and fibrous tissue are highly enriched from D10 to D14, reflecting the progressive stabilization and maturation of the atrial tissue (Fig. 6h). The overlap and evolution of key functional terms across different stages strongly support the functional consistency and developmental coherence of the atria during morphogenesis and structural remodelling.

### 3.6 SpatialModal reveals spatial patterns without histological image data

Many ST platforms, such as cyclic-ouroboros single-molecule fluorescence in situ hybridization (osmFISH) (Codeluppi *et al.* 2018), MERFISH (Chen *et al.* 2015), spatially resolved transcript amplicon readout mapping (STARmap) (Wang *et al.* 2018), and Stereo-seq (Chen *et al.* 2022a), generate high-resolution ST data without paired histological images. To demonstrate the robustness of SpatialModal in this context, we apply the model to datasets from these platforms and conduct a systematic evaluation, confirming its efficacy when relying solely on spatial coordinates and gene expression data.

**Figure 7:**
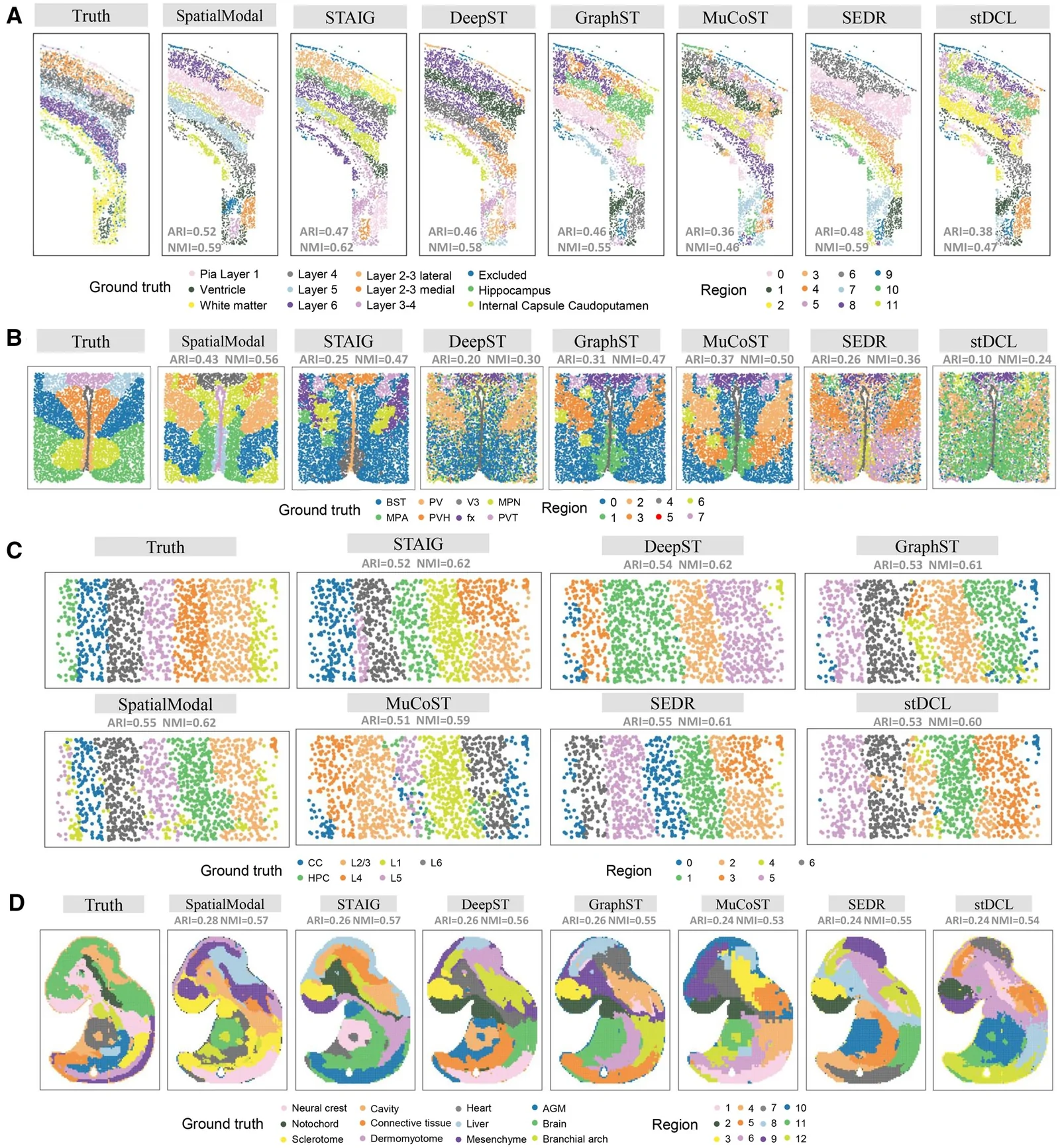
SpatialModal reveals spatial patterns without histological image data. (A) Annotations and SpatialModal clustering results for the mouse somatosensory cortex tissue section, with the corresponding ARI and NMI values. (B) Annotations and SpatialModal clustering results for the Bregma-0.14 tissue section, with the corresponding ARI and NMI values. (C) Annotations and SpatialModal clustering results for the mouse visual cortex tissue section, with the corresponding ARI and NMI values. (D) Annotations and SpatialModal clustering results for the mouse embryo tissue section, with the corresponding ARI and NMI values.

First, we apply SpatialModal to the mouse somatosensory cortex dataset generated by osmFISH. The results show that SpatialModal exhibits the highest identification accuracy in the clustering task (ARI = 0.52, NMI = 0.59), yielding regions that are also highly consistent with the ground truth annotations (Fig. 7a). Notably, SpatialModal accurately delineates Layers 2–3 into distinct medial (region 8) and lateral (region 3) sections, while exhibiting higher accuracy and resolution in identifying Layer 4 (region 0) and Layer 6 (region 7).

Next, we validate SpatialModal on the mouse preoptic area Bregma-0.14 tissue section from MERFISH. SpatialModal’s clustering performance substantially outperforms other methods, achieving the highest ARI (0.43) and NMI (0.56). It also demonstrates superior anatomical structure resolution, accurately identifying key brain regions such as the third ventricle (V3; region 7), paraventricular nucleus of the thalamus (PVT; region 4), and periventricular hypothalamic nucleus (PV; region 5) (Fig. 7b).

We then apply SpatialModal to the mouse visual cortex dataset from the STARmap platform. Clustering results show that SpatialModal achieves the highest scores on both ARI (0.55) and NMI (0.62). The identified regions 0, 6, and 5 are highly consistent with the manually annotated corpus callosum (CC), Layer 6 (L6), and Layer 5 (L5), respectively (Fig. 7c).

Finally, we validate SpatialModal on a mouse embryo dataset generated by Stereo-seq to further demonstrate its performance. When the number of clusters is set to match the ground truth, SpatialModal achieves the most accurate region partitioning, obtaining superior ARI (0.28) and NMI (0.57) (Fig. 7d).

## 4 Discussion

Recent advances in ST technologies permit simultaneous profiling of gene expression and high-resolution histology. By deeply integrating these intrinsically complementary dimensions, joint multimodal modelling overcomes the limitations of weak fusion approaches, achieving highly precise spatial domain identification and comprehensive characterization of the complex microenvironment (Li *et al.* 2026b). Here, we propose SpatialModal, a deep collaborative modelling framework that symmetrically integrates spatial multimodal data.

Comprehensive evaluations across a wide array of tissues and sequencing platforms confirm the exceptional robustness of SpatialModal. In complex human and mouse brain tissues, the framework faithfully identifies intricate cortical layers and anatomical regions. Meanwhile, SpatialModal successfully dissects highly heterogeneous disease microenvironments. In breast cancer tissues, it uncovers intra-regional subpopulations and elucidates underlying disease mechanisms. Similarly, the application to Alzheimer’s disease sections reveals aberrant cellular communication networks and region-specific molecular dysregulation. The model also accurately captures spatiotemporal developmental trajectories during embryonic heart development. Importantly, SpatialModal maintains highly competitive clustering performance even on unimodal datasets that completely lack histological images, highlighting its broad platform adaptability.

By generating highly robust latent representations, SpatialModal provides a reliable computational foundation for the systematic dissection of complex tissue heterogeneity. With the development of spatial multi-omics technologies, SpatialModal can be extended to a multi-omics framework. This extension will expand its biological applicability, providing a comprehensive computational framework for systematically characterizing tissue microenvironments and dissecting disease mechanisms.

## Supplementary Material

btag540_Supplementary_Data

## Data Availability

The data underlying this article are publicly available on Zenodo at https://doi.org/10.5281/zenodo.18220735. The SpatialModal source code is openly accessible through GitHub at https://github.com/xingyili/SpatialModal, and the version used to generate the results reported in this study has been permanently archived on Zenodo at https://doi.org/10.5281/zenodo.21264356
