# Supplementary material for: Deciphering spatial heterogeneity by multimodal spatial transcriptomics modelling with SpatialModal
Source: Bioinformatics. 2026 Jul 21;42(8):btag540. doi: 10.1093/bioinformatics/btag540 (PMC13453310; doi:10.1093/bioinformatics/btag540)
Supplement: Supplementary file 1 [file btag540_supplementary_data.pdf]

Supporting information to

Deciphering spatial heterogeneity by multimodal spatial transcriptomics modeling with SpatialModal

Supplementary Notes

Supplementary Note S1: Evaluation metrics

For datasets with manual annotations, the Adjusted Rand Index (ARI)[8] and Normalized Mutual Information (NMI)[9] serve as evaluation metrics. For datasets without ground-truth labels, the Silhouette Coefficient (SC)[10] and Davies-Bouldin Index (DB)[11] are used as the evaluation metrics.

- **ARI**

ARI, an adjusted version of the Rand Index, measures the similarity between clustering results and true labels, accounting for random effects. The formula is:

$$ARI = \frac{RI - E[RI]}{\max(RI) - E[RI]}$$

where RI is the Rand Index, calculated as:

$$RI = \frac{a + b}{C_n^2}$$

where, a represents the number of sample pairs from the same class assigned to the same cluster, b denotes the number of sample pairs from different classes assigned to different clusters, and C_n^2 is the total number of sample pairs. ARI ranges from [-1, 1], with higher values indicating better clustering performance.

- **NMI**

NMI measures the consistency between clustering results and true labels through mutual information, which is normalized by the entropy of the cluster assignments. The formula is:

$$NMI = \frac{2I(Y, C)}{H(Y) + H(C)}$$

where $I(Y, C)$ is the mutual information between true labels Y and clustering results C. $H(Y)$ and $H(C)$ are the entropies of true labels and clustering results, respectively. NMI ranges from [0, 1], with higher values reflecting superior clustering quality.

- **SC**

SC evaluates the cohesion and separation of each sample within its cluster. The formula for a sample i is:

$$s(i) = \frac{b(i) - a(i)}{\max\{a(i), b(i)\}}$$

where $a(i)$ is the average distance from sample i to other samples in the same cluster, and $b(i)$ is the average distance from sample i to all samples in the nearest different cluster. The overall Silhouette Score is the average of $s(i)$ across all samples:

$$Silhouette\ score = \frac{1}{N}\sum_{i=1}^{N} s(i)$$

The score ranges from [-1, 1], with higher values indicating better clustering.

- **DB**

DB measures the ratio of within-cluster cohesion to between-cluster separation:

$$R_{ij} = \frac{s_i + s_j}{d_{ij}}$$

where s_i is the average distance from samples in cluster C_i to its centroid, s_j is the average

distance from samples in cluster C_j to its centroid, and d_{ij} is the distance between the centroids of clusters C_i and C_j. For each cluster C_i, the worst-case R_{ij} is selected, and the score is averaged across all clusters:

$$Davies - bouldin\ score = \frac{1}{k}\sum_{i=1}^{k} \max_{j \neq i} R_{ij}$$

The score ranges from [0, $+\infty$], with lower values indicating better clustering performance.

Supplementary Note S2: Downstream task tools

- **K-means clustering**

K-means clustering is a distance-based algorithm that partitions data points into K clusters by minimizing the within-cluster sum of squared distances to cluster centroids. Using the scikit-learn implementation, we apply K-means to the latent embeddings for initial clustering. To enhance spatial coherence, we adjust cluster assignments by majority voting among 50 nearest neighbors in Euclidean space.

Datasets applied: Human DLPFC datasets, human breast cancer dataset, mouse somatosensory cortex, and mouse primary visual cortex datasets.

- **Model-based clustering (Mclust)**

Model-based clustering assumes data generation from a Gaussian mixture model, where parameters are estimated via expectation-maximization to assign cluster labels. Using the mclust R package, we fit ellipsoidal clusters with equal covariance structures.

Datasets applied: Mouse anterior/posterior/coronal brain datasets, Normal and Alzheimer's disease human prefrontal cortex datasets, chicken heart datasets, mouse hypothalamic, and mouse embryo.

- **Uniform manifold approximation and projection (UMAP)[12]**

UMAP is a nonlinear dimensionality reduction technique that preserves local and global topological structures by modeling high-dimensional data as a weighted graph. We apply UMAP for 2D visualization of latent embeddings to elucidate spatial domain relationships.

- **Partition-based graph abstraction (PAGA)[13]**

PAGA constructs simplified graphs to quantify connectivity between annotated clusters, enabling trajectory inference by resolving transitional states and lineage relationships.

- **Diffusion pseudotime (DPT)[14]**

DPT computes pseudotemporal ordering using diffusion operators derived from cell similarity graphs. Starting from user-defined root cells, cumulative diffusion distances are calculated via random walks, implemented using *scanpy.tl.diffmap* and *scanpy.tl.dpt*.

- **Ripley's L function[15]**

Ripley's L-function evaluates spatial clustering or dispersion of point patterns. The L-function is defined as:

$$L(r) = \sqrt{K(r)/\pi}$$

where $K(r)$ estimates the average number of neighboring points within radius *r*. Values for which $L(r) > r$ indicate clustering, those for which $L(r) < r$ indicate dispersion, and those for which $L(r) \approx r$ indicate randomness.

- **CellChat[16]**

CellChat analysis was performed using the CellChat R package. In this study, CellChat was run in spatial mode, and the spatial domain labels were used as the grouping information. The spatial coordinates of spots were used to construct the spatial location matrix, which was provided to *createCellChat()* together with the Visium spatial scale factors. Communication probabilities were calculated using *computeCommunProb()* with spatial geometric distance constraints. The parameters were set as follows: *type = "truncatedMean"*, *trim = 0.1*, *distance.use = TRUE*, *interaction.range = 150*, *scale.distance = 0.1*, *contact.dependent = TRUE*, and *contact.range = 50*. The inferred communications were then filtered using *filterCommunication(cellchat, min.cells = 10)*. Pathway-level communication probabilities were calculated using

computeCommunProbPathway(), and the communication network was aggregated using *aggregateNet()*. Outgoing communication patterns were identified using *identifyCommunicationPatterns(cellchat, pattern = "outgoing", k = 4)*.

- **Identification of differentially expressed genes (DEGs)**

We identify spatial domain-specific DEGs using *scanpy.tl.rank_genes_groups* with the Wilcoxon rank-sum test. Genes are ranked by absolute z-scores ($|z| > 3$), adjusted p-values (Benjamini-Hochberg FDR < 0.05), and log-fold changes ($|\text{logFC}| > 1$).

- **GO enrichment analysis**

GO enrichment analysis identifies statistically overrepresented biological processes, molecular functions, and cellular components among DEGs using hypergeometric testing. Significantly enriched terms (p-value < 0.05) are reported.

- **Waddington-OT[17]**

Waddington-OT analysis was performed using the Waddington-OT package to infer developmental transition relationships among SpatialModal-inferred spatial domains across chicken heart development. Gene expression profiles from the D7, D10, and D14 sections were used as input, and only genes shared across all three stages were retained to ensure comparability across time points. The three stages were treated as ordered developmental time points, with D7, D10, and D14 encoded as 0, 1, and 2, respectively. The initial growth rate was set uniformly to 1 for all spots, without incorporating additional proliferation priors.

Optimal transport maps were inferred between consecutive developmental stages with the following parameter settings: $\epsilon = 0.05$, $\epsilon_0 = 1$, $\lambda_1 = 0.1$, $\lambda_2 = 5$, and the number of growth-rate learning iterations was set to 3. The inferred spot-level transport probabilities were summarized at the spatial-domain level by aggregating transitions between groups defined by developmental stage and SpatialModal-inferred domain labels. To obtain comparable and conservative estimates of domain-level transition strengths, each transition matrix was row-normalized by source domains and column-normalized by target domains, and the element-wise minimum of the two normalized matrices was used as the final transition weight matrix.

- **Cell enrichment score**

For datasets with cell type annotations, we further calculated the cell enrichment score for each spatial domain. Specifically, we grouped spots by annotated cell type and identified spatial domain to construct a cell type × spatial domain count matrix. Each element $C(i,j)$ represents the number of spots/cells of cell type i in spatial domain j. To compare the relative enrichment of each cell type across spatial domains while accounting for differences in overall cell type abundance, we performed row-wise z-score normalization for each cell type across all spatial domains. The cell enrichment score for cell type i in spatial domain j was calculated as:

$$E(i,j) = \frac{C(i,j) = \text{mean}(C(i,*))}{\text{std}(C(i,*))}$$

where, $C(i,*)$ denotes the count vector of cell type i across all spatial domains. The resulting $E(i,j)$ was used as the cell enrichment score in downstream analyses.

Supplementary Note S3: Multi-slice joint graph construction

To facilitate the joint training of multiple tissue slices while preserving their unique spatial topologies, SpatialModal models the dataset as a global disjoint graph. Let $S = \{S_1, S_2, \cdots, S_K\}$ denote the set of K spatial omics sections. For the k-th section ($k \in [1, K]$), we denote the gene expression matrix as $X_k \in \mathbb{R}^{N_k \times D_g}$, the morphological image feature matrix as $I_k \in \mathbb{R}^{N_k \times D_v}$, and the spatial adjacency matrix as $A_k \in \mathbb{R}^{N_k \times N_k}$, where N_k represents the number of spots in section k.

Instead of processing each section individually, we unify the feature spaces by vertically stacking the representations. This constructs a global gene expression matrix X and a global image feature matrix I, defined as:

$$X = \begin{bmatrix} X_1 \\ X_2 \\ \vdots \\ X_K \end{bmatrix}, I = \begin{bmatrix} I_1 \\ I_2 \\ \vdots \\ I_K \end{bmatrix}$$

In the spatial domain, we rigorously prohibit inter-slice message passing to reflect the physical isolation of different tissue sections. We formulate the global adjacency structure A_{global} as a block-diagonal matrix, which corresponds to the disjoint union of individual slice graphs:

$$A_{global} = \mathrm{diag}(A_1, A_2, \cdots, A_K) = \begin{bmatrix} A_1 & 0 & \cdots & 0 \\ 0 & A_2 & & 0 \\ \vdots & & \ddots & \vdots \\ 0 & 0 & \cdots & A_K \end{bmatrix}$$

Under this formulation, SpatialModal takes X, I and A_{global} as input.

Supplementary Note S4: Spatial mapping-based denoising

To enhance the biological consistency of denoised gene expression data, we introduce a spatial mapping-based denoising module inspired by the manifold learning strategy in STAGE[20]. This module utilizes an MLP-based encoder-decoder architecture, where the encoder projects the input latent features Z_{latent} into a low-dimensional intermediate representation Z_{mid}, and the decoder subsequently reconstructs the gene expression profile X_{rec}. Crucially, we impose a spatial constraint by explicitly aligning Z_{mid} with the normalized physical coordinates $P(\cdot)$. For single-slice data, $Z_{\mathrm{mid}} \in \mathbb{R}^{N\times 2}$ is supervised using 2D coordinates via the following loss:

$$\mathcal{L}_{\mathrm{map}} = \frac{1}{N}\sum_{i=1}^{N} \left|\left|Z_{\mathrm{mid},i} - P(x_i, y_i)\right|\right|_1$$

For multi-slice joint analysis, $Z_{mid} \in \mathbb{R}^{N\times 3}$ is designed to accommodate the slice index z, and is formulated as follows:

$$\mathcal{L}_{\mathrm{map}} = \frac{1}{N}\sum_{i=1}^{N} \left|\left|Z_{\mathrm{mid},i} - P(x_i, y_i, z_i)\right|\right|_1$$

The module is trained end to end by minimizing the composite objective:

$$\mathcal{L} = \alpha\mathcal{L}_{\mathrm{map}} + \beta(\frac{1}{N}\sum_{i=1}^{N} ||X_{\mathrm{rec},i} - X_{g,i}||_2^2)$$

where α and β are hyperparameters. We employ the Adam optimizer with a learning rate of 0.001 and a step-based scheduler for optimization.

Supplementary Note S5: Ablation analysis of the cross-modal masked reconstruction strategy
To further validate the contribution of the cross-modal masked reconstruction strategy in SpatialModal, we performed two additional ablation analyses on three representative DLPFC sections, including 151509, 151672, and 151674.

First, we evaluated whether this mechanism regulates the contribution of different modalities to the VGAE fusion representation and prevents the fused representation from being dominated by a single modality. To this end, we compared Full SpatialModal with SpatialModal w/o cross-modal masked reconstruction and used linear centered kernel alignment (linear CKA)[28], to quantify the similarity between the VGAE fusion output and the gene-specific or image-specific representations. As shown in Supplementary Table S4, Full SpatialModal achieved an average image/gene ratio of 0.821, whereas removing cross-modal masked reconstruction reduced this value to 0.366. Meanwhile, after removing this mechanism, the similarity between the VGAE output and the gene-specific representation increased from 0.575 to 0.850, whereas the similarity with the image-specific representation decreased from 0.473 to 0.308. These results indicate that, without cross-modal masked reconstruction, the VGAE fusion representation tends to be dominated by the gene modality. In contrast, the full model enables histological image information to participate more effectively in fusion representation learning, thereby regularizing the multimodal fusion process.

Second, we further evaluated the contribution of cross-modal replacement and mask reconstruction loss to spatial domain identification. As shown in Supplementary Table S5, Full SpatialModal achieved the highest ARI on all three DLPFC sections, with an average ARI of 0.646. In comparison, SpatialModal w/o cross-modal replacement, SpatialModal w/o mask reconstruction loss, and SpatialModal with random feature masking achieved average ARI values of 0.401, 0.362, and 0.428, respectively. These results demonstrate that the complete cross-modal masked reconstruction mechanism consistently improves spatial domain identification.

Supplementary Note S6: Hyperparameter sensitivity analysis

To evaluate the robustness of SpatialModal under different hyperparameter settings, we performed a hyperparameter sensitivity analysis on three representative DLPFC sections, including 151509, 151672, and 151674. For each analysis, one hyperparameter was varied at a time while all other settings were kept unchanged. The clustering performance was evaluated using ARI, and the results are summarized in Supplementary Figure S11.

We examined key hyperparameters from different components of SpatialModal, including the fusion dimension d_2, the cross-modal perturbation ratio p_{perturb}, the temperature parameter τ, edge dropping rates, feature masking rates, loss weights for masked reconstruction, prototype-level contrastive learning, spot-level contrastive learning, and VGAE regularization, as well as the spatial neighborhood number used for graph construction and the prototype number used in cluster-level contrastive learning.

Overall, SpatialModal showed stable performance across a broad range of hyperparameter settings. For the fusion dimension d_2, the model maintained competitive performance under different latent dimensions, indicating that the learned representation was not highly sensitive to the exact fusion dimensionality. For graph and feature perturbation in spot-level contrastive learning, moderate perturbation strengths generally led to better performance, whereas overly strong perturbations tended to degrade clustering accuracy, suggesting that excessive corruption may disrupt biologically meaningful spatial and transcriptional structures.

The sensitivity analysis of loss weights further showed that SpatialModal was robust to a range of reconstruction and contrastive learning weights. In particular, the masked reconstruction and prototype-level contrastive terms contributed to stable spatial domain identification across the tested settings. For the spot-level contrastive loss, however, very small or overly large weights led to decreased ARI values, indicating that an appropriate balance between local contrastive regularization and other training objectives is important for effective representation learning. The VGAE-related weight also showed generally stable behavior across multiple settings, supporting the robustness of the multimodal fusion module.

We also evaluated the spatial neighborhood number used for graph construction and the prototype number used in cluster-level contrastive learning. The results showed that SpatialModal achieved relatively stable performance under different graph neighborhood settings, while overly sparse or overly dense neighborhood structures could reduce clustering accuracy. For cluster-level contrastive learning, using a small number of prototypes provided effective coarse-grained contrastive regularization, whereas increasing the prototype number did not consistently improve performance.

Supplementary Figures

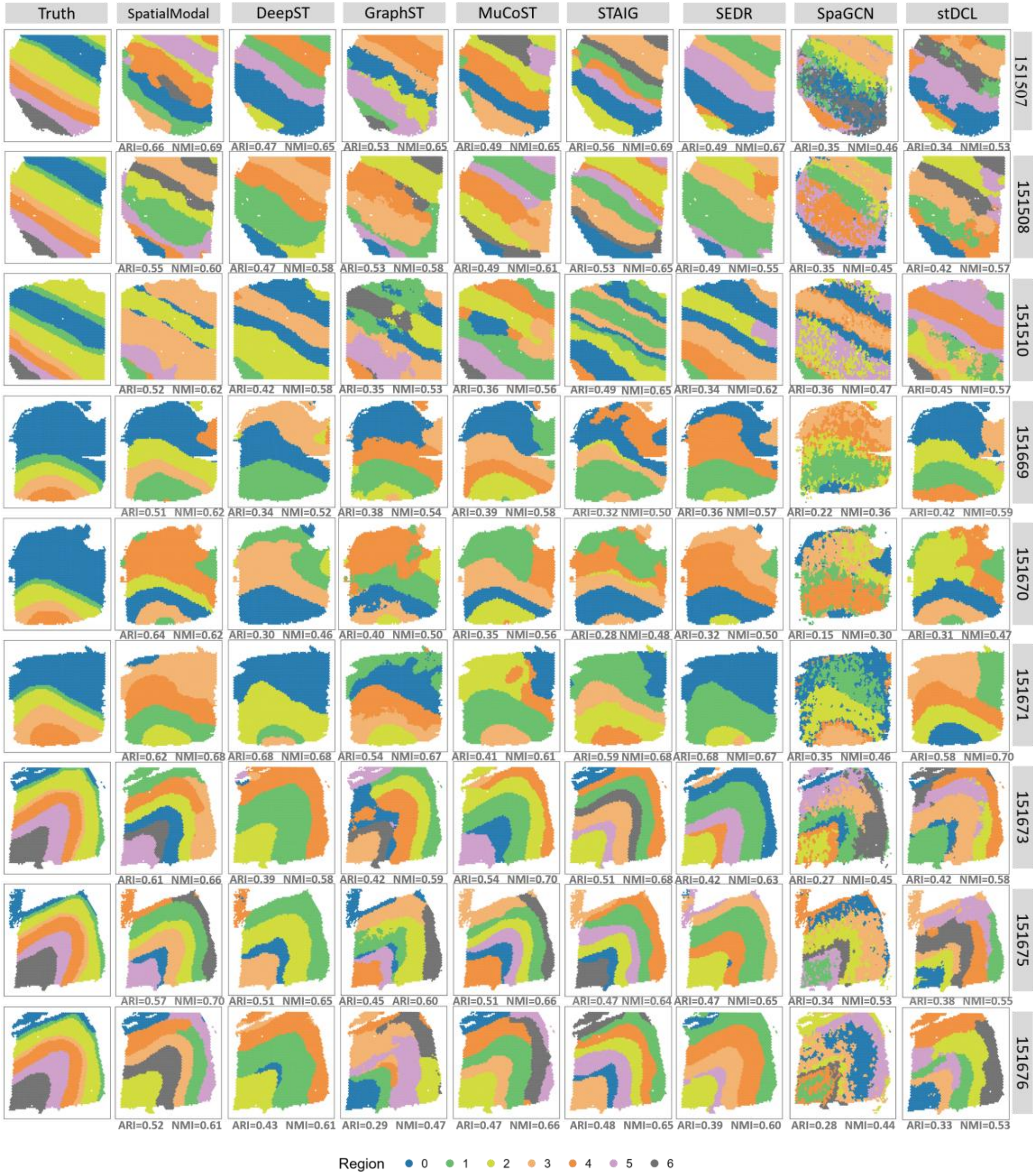

Supplementary Figure S1. Evaluation on the DLPFC dataset. Spatial clustering results of each method on nine additional slices, alongside the ground-truth annotations and quantitative benchmarking metrics (ARI and NMI).

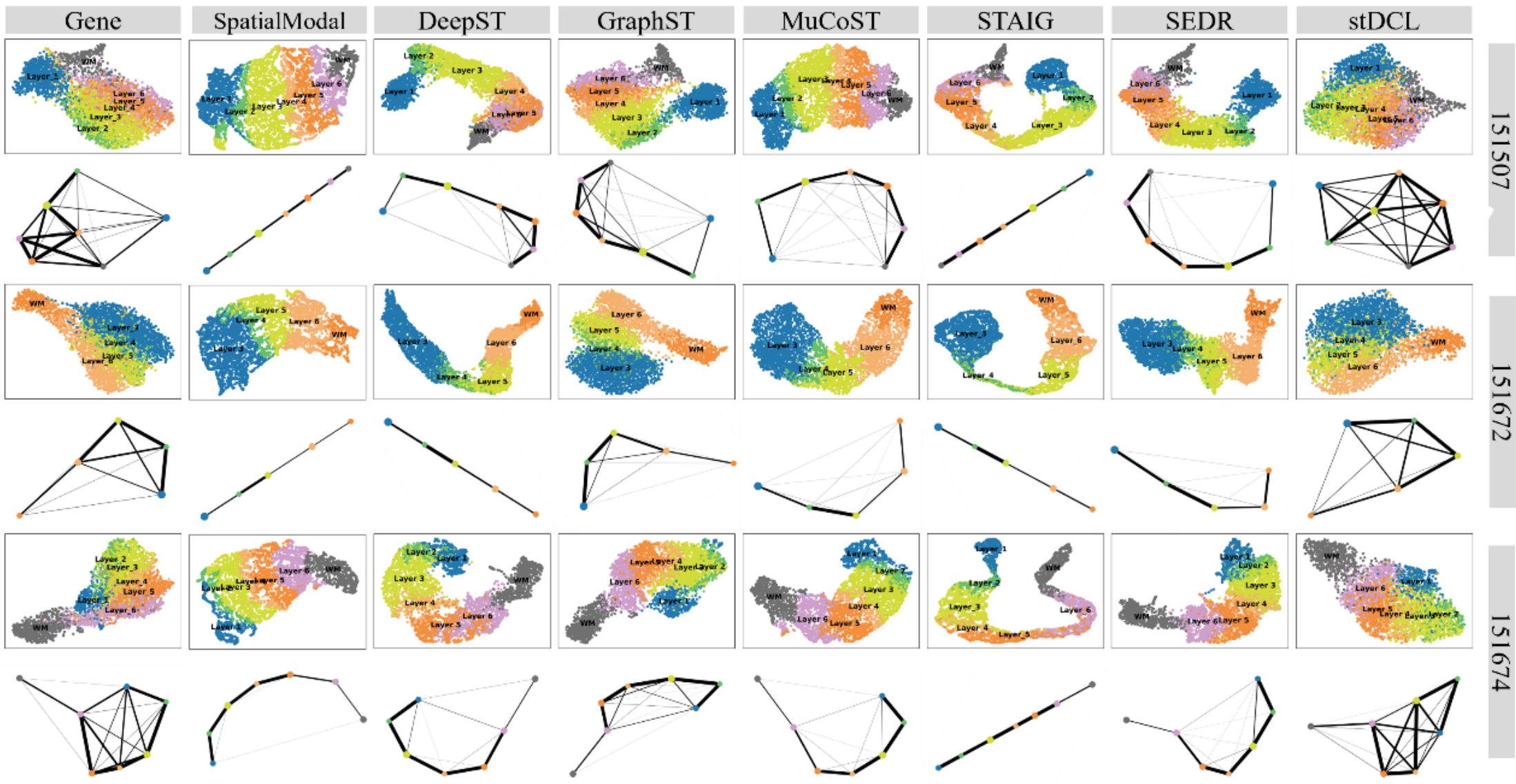

Supplementary Figure S2. UMAP and PAGA plots on three DLPFC slices. This figure displays UMAP and PAGA plots for the three representative DLPFC slices (151507, 151672, and 151674) discussed in the main text. For each slice, the visualizations are derived from the latent representations learned by SpatialModal and six other benchmarking methods. The UMAP plots illustrate the separation of spatial domains in low-dimensional space, while the PAGA graphs quantify the structural connectivity and transitional relationships among the identified clusters. Baseline visualizations generated directly from raw gene expression data (labeled "Gene") are provided to demonstrate improvements in representation learning achieved by the computational models.

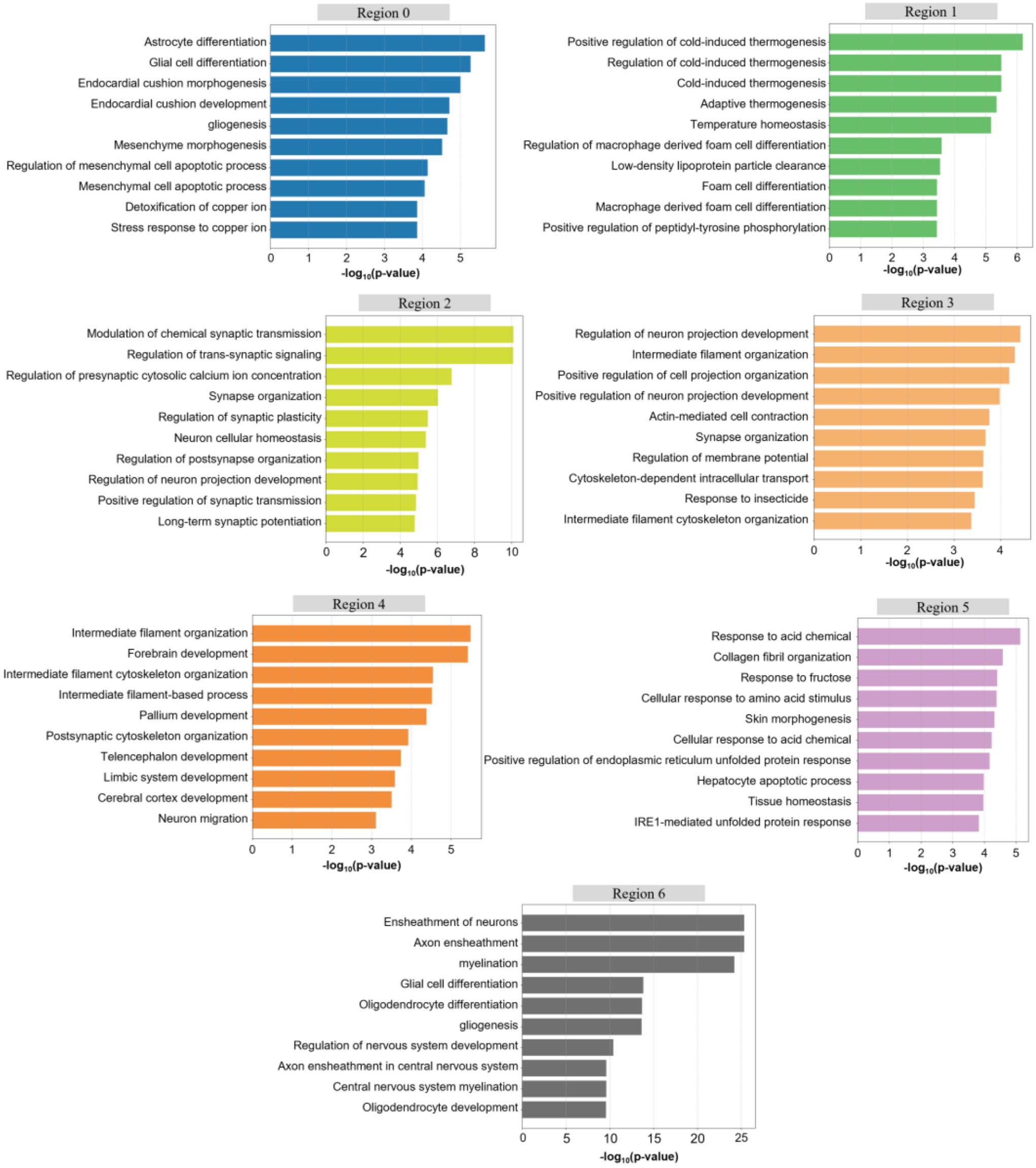

Supplementary Figure S3. GO enrichment analysis results for slice 151509 in the DLPFC dataset. The bar plots illustrate the top 10 GO terms for each identified region by SpatialModal.

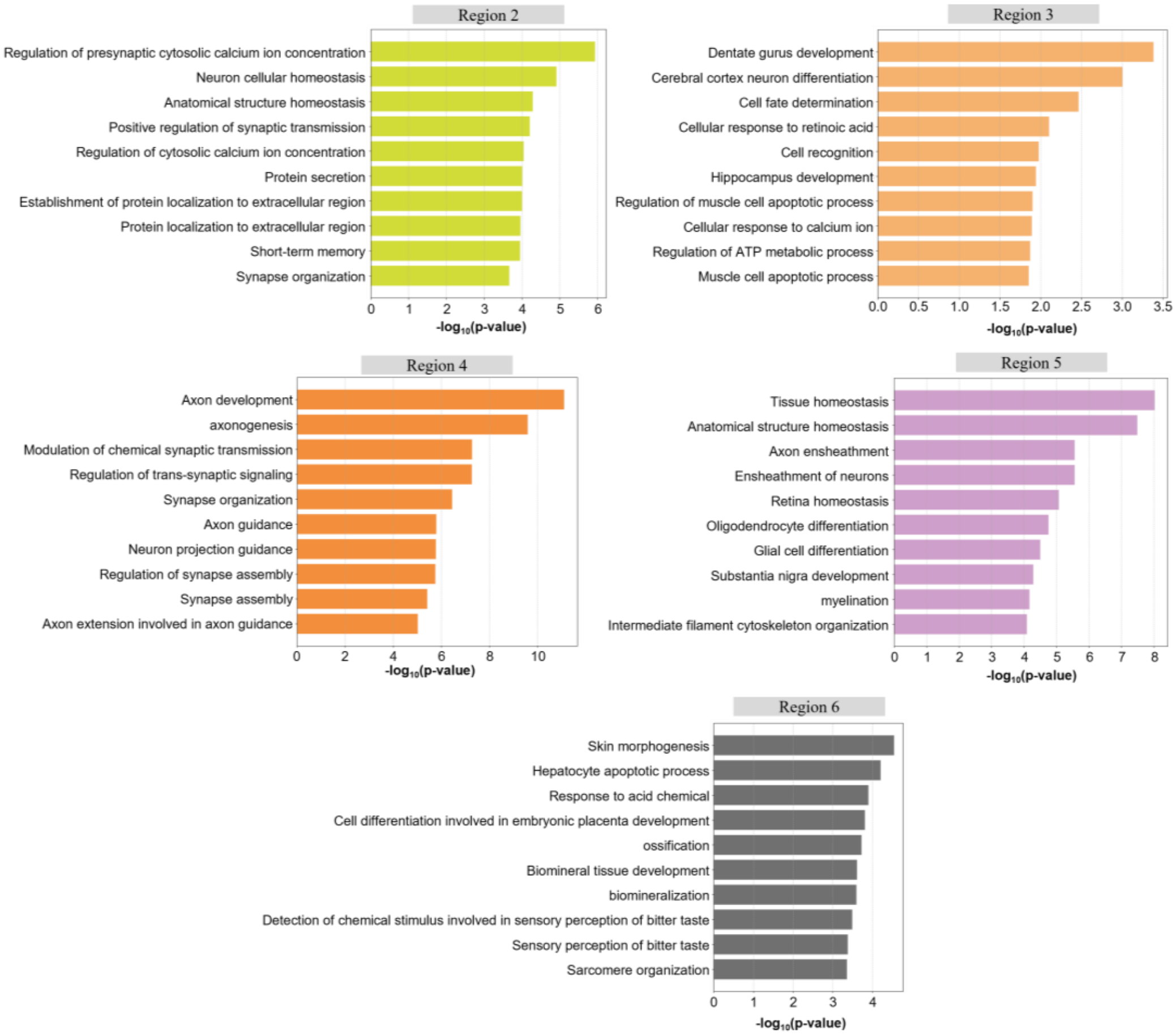

Supplementary Figure S4. GO enrichment analysis results for slice 151672 in the DLPFC dataset. The bar plots illustrate the top 10 GO terms for each identified region by SpatialModal.

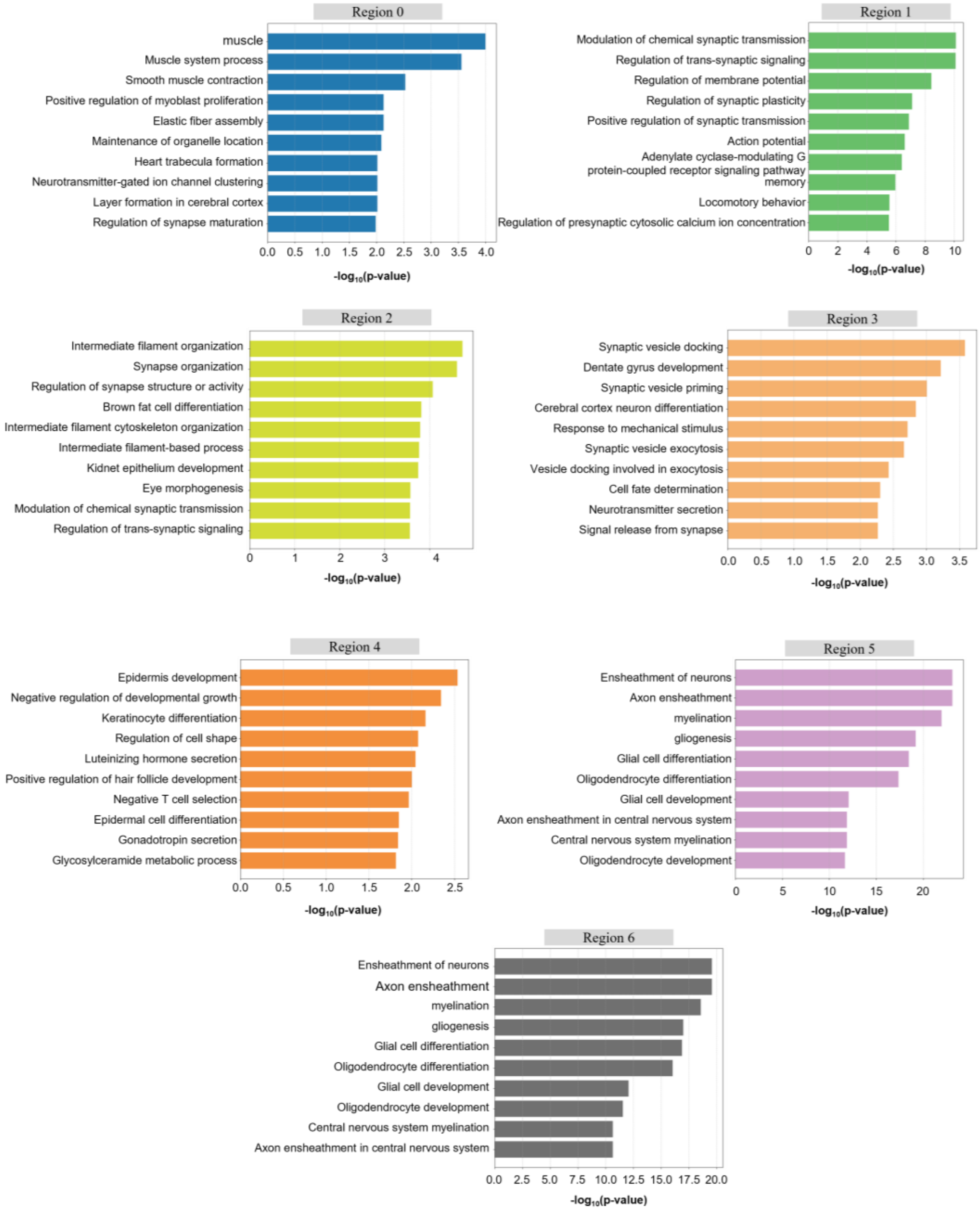

Supplementary Figure S5. GO enrichment analysis results for slice 151674 in the DLPFC dataset. The bar plots illustrate the top 10 GO terms for each identified region by SpatialModal.

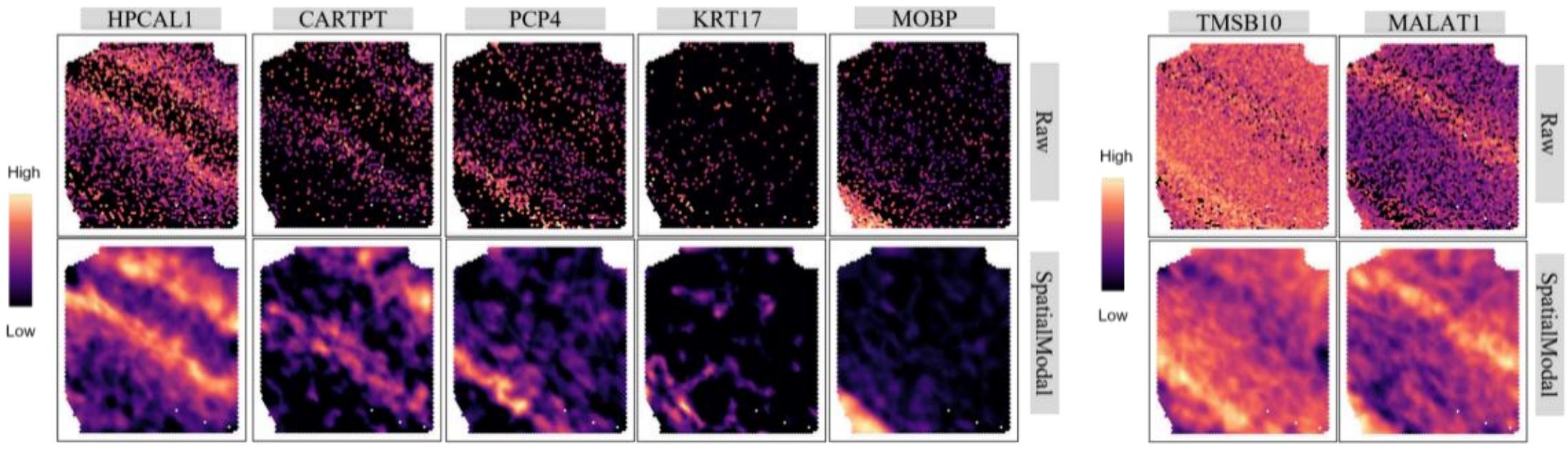

Supplementary Figure S6. Evaluation of Gene Denoising. Raw and reconstructed expression patterns of the genes (*HPCAL1, CARTPT, PCP4, KRT17, MOBP, TMSB10, MALAT1*).

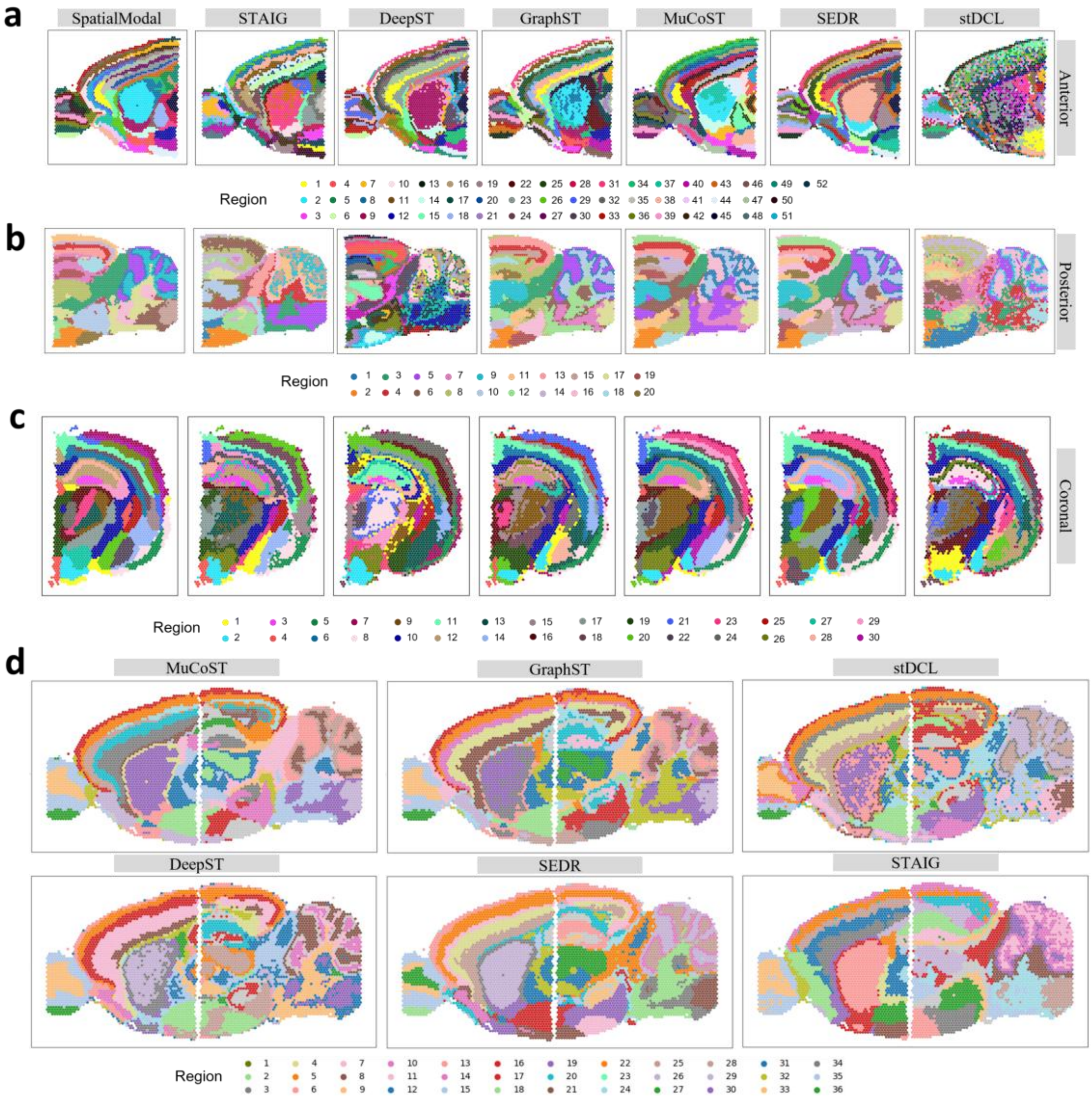

Supplementary Figure S7. Evaluation on the mouse brain dataset. a. Spatial clustering results of each method on sagittal anterior slice. b. Spatial clustering results of each method on sagittal posterior slice. c. Spatial clustering results of each method on coronal slice. d. Joint spatial clustering results of each method on sagittal anterior and posterior slices.

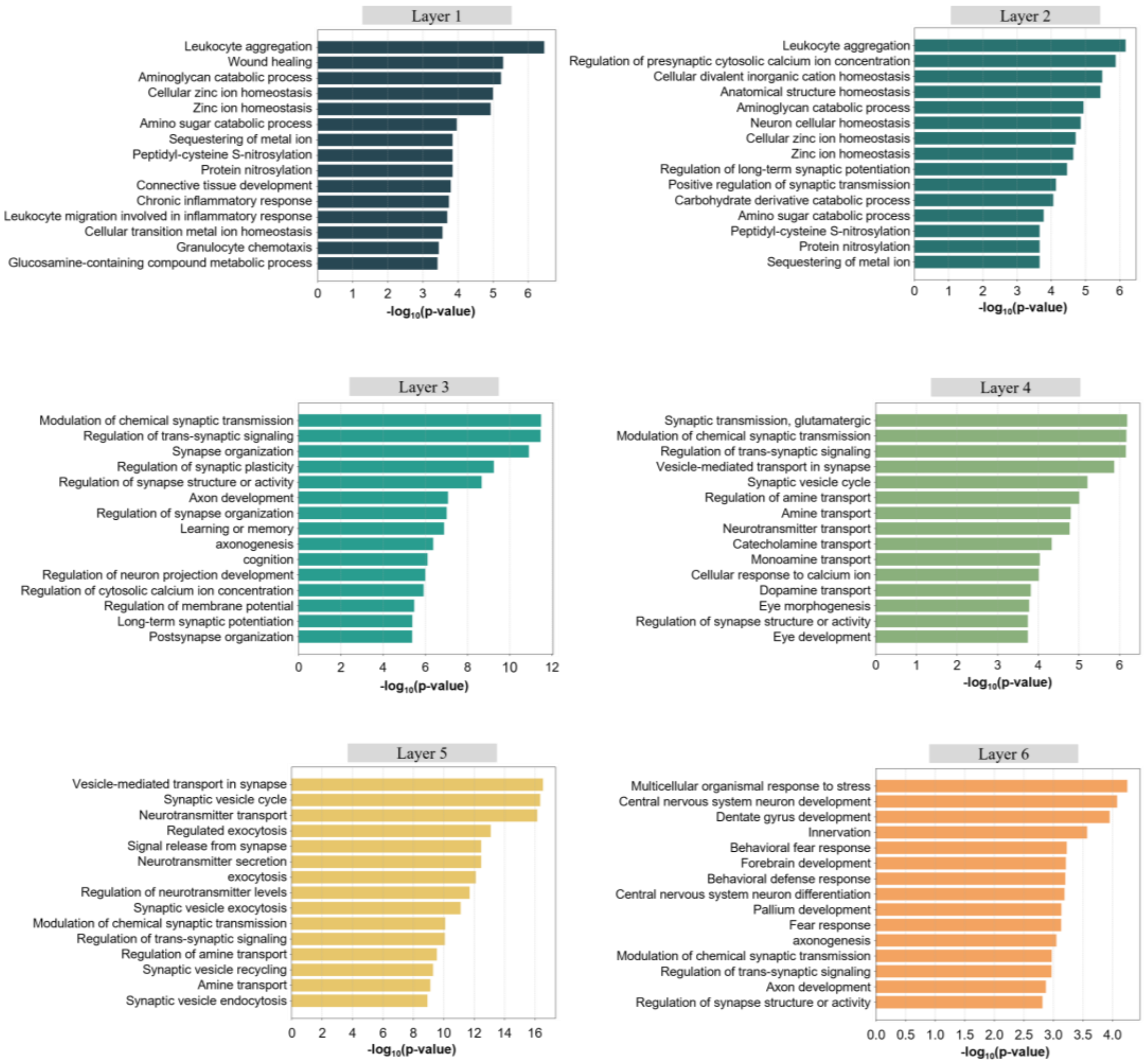

Supplementary Figure S8. GO enrichment analysis results in the human MTG data. The bar plots illustrate the top 10 GO terms for each identified region by SpatialModal.

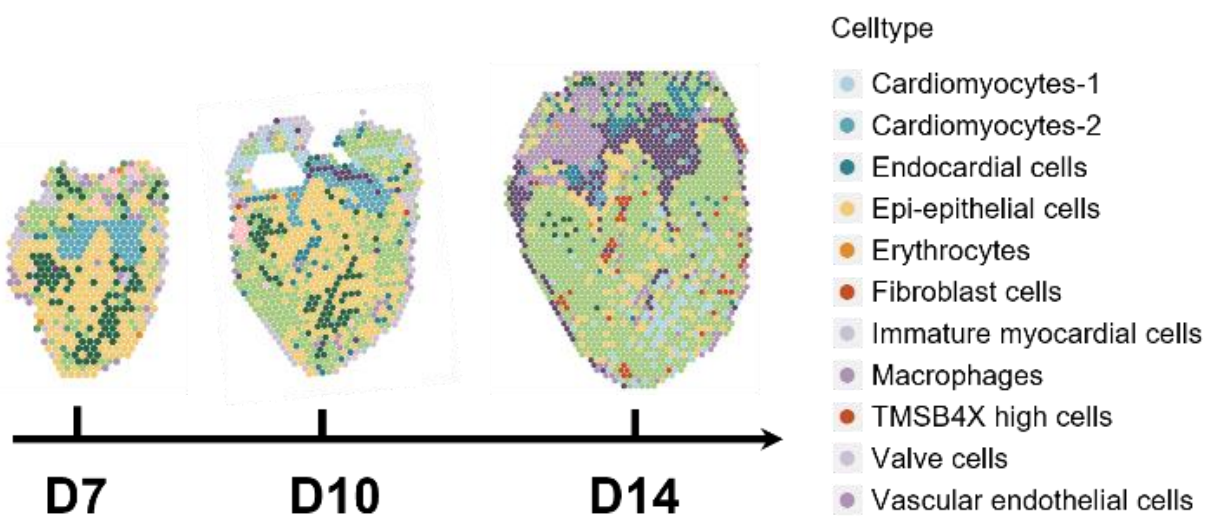

Supplementary Figure S9. Cellt-ype annotations in chick hearts data at three different developmental stages.

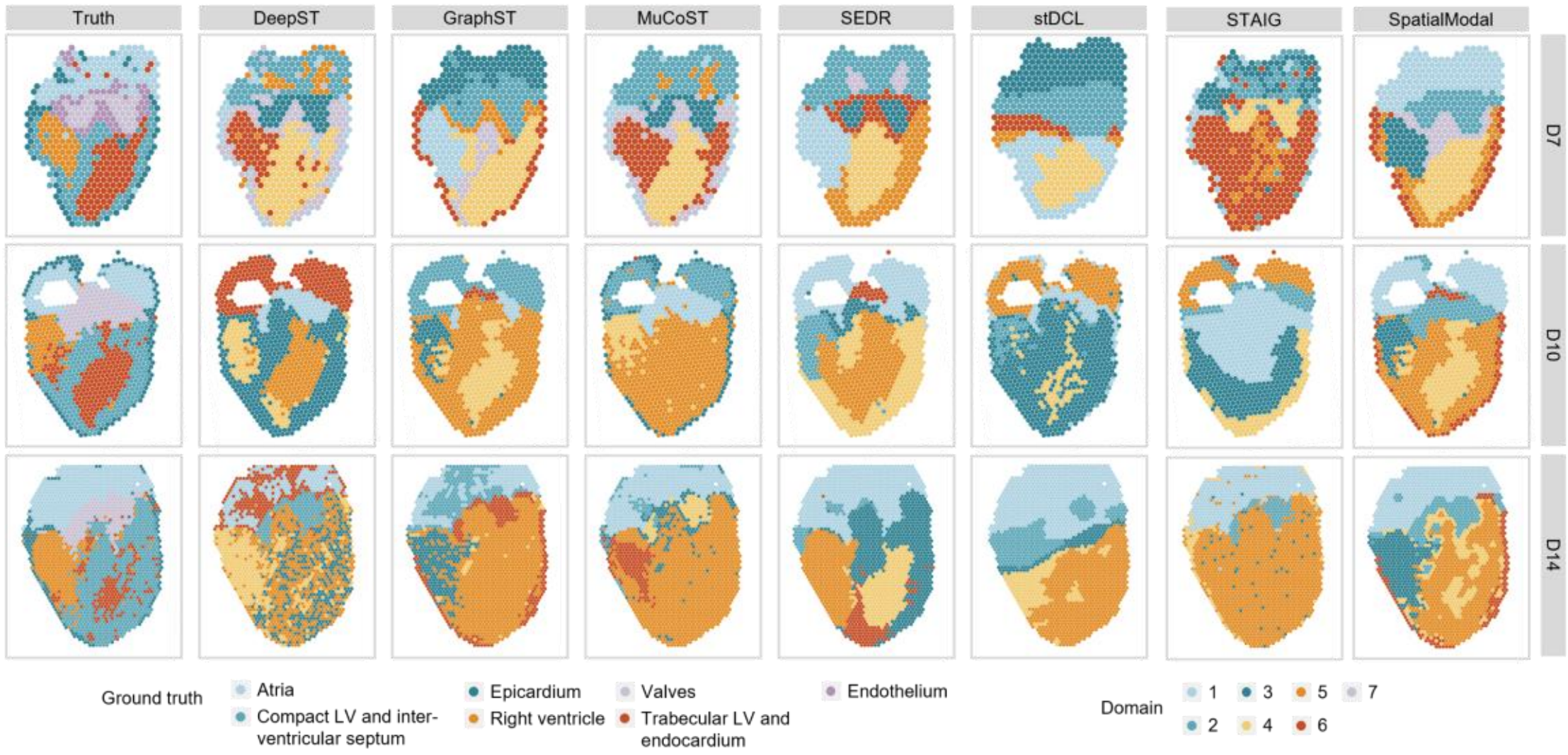

Supplementary Figure S10. Evaluation on the chick heart dataset. Spatial clustering results of each method on chick heart slices at three distinct developmental stages, alongside the corresponding annotations.

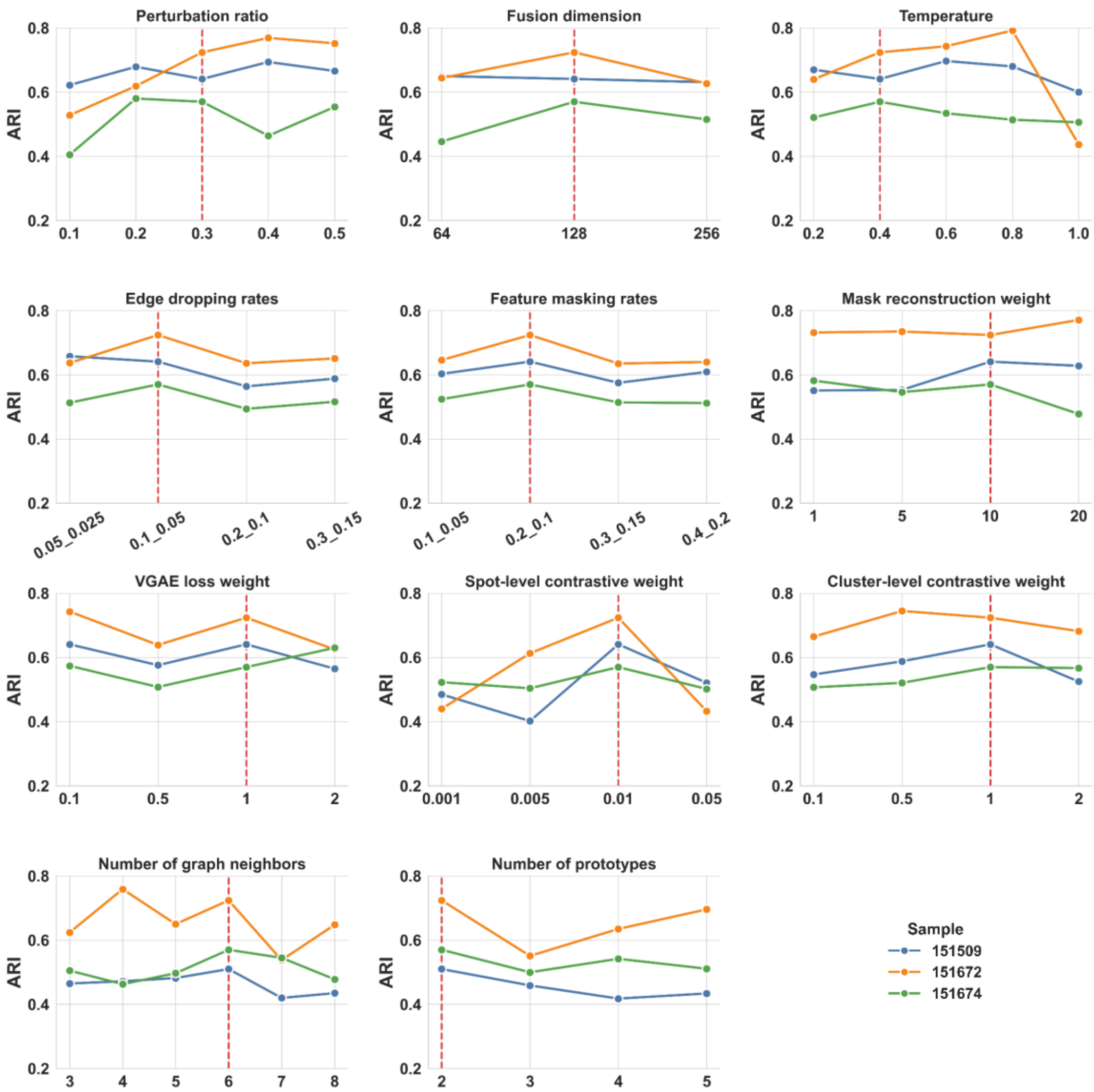

Supplementary Figure S11. Hyperparameter sensitivity analysis of SpatialModal. Line plots show the ARI values of SpatialModal on three representative DLPFC sections under different hyperparameter settings. In each panel, one hyperparameter was varied while the others were fixed. Red dashed lines indicate the default values used in the main experiments.

Supplementary Tables

Supplementary Table S1. Methodological comparison between SpatialModal and benchmark methods. This table summarizes the input modalities, graph/structure modeling strategies, and image feature usage of SpatialModal and seven benchmark methods. All benchmark methods were implemented using the default settings described in their original publications.

**Method**	**Input modalities**	**Graph / structure modeling strategy**	**Image feature usage**	**Main characteristics**
SpatialModal	Gene, spatial coordinates, images	Constructs a spatial adjacency graph based on spatial coordinates, which is used for multimodal GCN encoding, VGAE fusion, and graph contrastive learning.	Uses ResNet-152 to extract deep morphological features from H&E image patches; image features are treated as an independent modality for representation learning.	Integrates multimodal information through hierarchical multimodal representation, VGAE fusion, cross-modal masked reconstruction, and dual-level contrastive learning.
DeepST[5]	Gene, spatial coordinates, images	Constructs a spatial adjacency graph based on spatial coordinates; expression similarity and morphological similarity are mainly used to generate an augmented gene expression matrix.	Uses a pretrained CNN to extract morphological features from H&E image tiles, which are used to calculate morphological similarity and augment gene expression.	Integrates spatial proximity, expression similarity, and morphological similarity through spatial data augmentation before graph representation learning.
SpaGCN[6]	Gene, spatial coordinates, images	Constructs a histology-aware weighted spatial graph, with edge weights jointly determined by spatial distance and image information.	Uses RGB information from H&E images to construct histology-derived coordinates, which are used to adjust spot-to-spot distances and edge weights.	Uses GCN to aggregate gene expression from neighboring spots for spatial domain identification and spatially variable gene detection.
STAIG[7]	Gene, spatial coordinates, images	Constructs a spatial adjacency graph based on spatial distances and generates augmented graph views through image-guided edge removal.	Uses BYOL to extract H&E image embeddings, which guide graph augmentation and debiased contrastive learning.	An image-aided graph contrastive learning framework for spatial domain identification and alignment-free multi-slice integration.

GraphST[1]	Gene, spatial coordinates	Constructs a spatial adjacency graph based on spatial coordinates and generates corrupted graphs for contrastive learning while preserving graph topology.	Does not use histological image features as model input for spatial domain identification.	Uses graph self-supervised contrastive learning to learn gene expression and local spatial context representations.
SEDR[4]	Gene, spatial coordinates	Constructs a spatial adjacency graph based on spatial coordinates and learns spatial embeddings through VGAE.	Does not use histological image features.	Combines a masked self-supervised deep autoencoder and VGAE to jointly learn gene representation and spatial embedding.
MuCoST[2]	Gene, spatial coordinates	Constructs a spatial adjacency graph, co-expression graph, and shuffled graph for multi-view graph modeling.	Does not use histological image features.	Uses multi-view graph contrastive learning to model both local spatial adjacency dependency and nonlocal gene co-expression dependency.
stDCL[3]	Gene, spatial coordinates	Constructs a spatial-expression joint graph by combining spatial adjacency and expression similarity.	Does not use histological image features.	Uses a graph convolutional autoencoder, space-aware contrastive learning, and cluster-level feature contrastive learning to improve spatial domain identification.

Supplementary Table S2. Description of all ST datasets used in this study.

Platform	Tissue	Section	#Spots/Bins	Source
10x Visium	Human dorsolateral prefrontal cortex (DLPFC)[21,22]	151507	4226	https://github.com/LieberInstitute/spatialDLPFC
		151508	4384	
		151509	4789	
		151510	4634	
		151669	3661	
		151670	3498	
		151671	4110	
		151672	4015	
		151673	3639	
		151674	3673	
		151675	3592	
		151676	3460	
	Mouse brain	Mouse Brain Section (Sagittal-anterior)	2695	https://www.10xgenomics.com/resources/datasets
		Mouse Brain Section (Sagittal-posterior)	3355	
		Mouse Brain Section(Coronal)	6050	
	Human breast	Human Breast Cancer Section 1	3798	
	Chicken heart[23]	D7	535	https:// doi.org/10.5281/zenodo.4517120
		D10	969	
		D14	1967	
	Human Middle Temporal Gyrus (MTG)[22]	CT	4701	https://www.ncbi.nlm.nih.gov/geo/query/acc.cgi?acc=GSE149457
		AD	4832	
osmFISH	mouse somatosensory cortex[24]	X	4839	http://linnarssonlab.org/osmFISH/
MERFISH	mouse hypothalamic[25]	X	5926	
STARmap	mouse primary visual cortex[26]	V1	1020	https://drive.google.com/drive/folders/1I1nxheWlc2RXSdiv24dex3YRaEh780my
Stereo-seq	mouse embryo[27]	E9.5_E1S1	5913	https://db.cngb.org/stomics/mosta/

Supplementary Table S3. Hyperparameter settings of SpatialModal.

**Module**	**Hyperparameter**	**Symbol**	**Value**
Feature projection	Projected dimension	d_1	512
Hierarchical representation	Fusion dimension	d_2	128
Cross-modal perturbation	Perturbation ratio	$p_{perturb}$	0.3
VGAE	Latent dimension	latent dim	16
Spot-level contrastive learning	Edge dropping rate	p_{edge}	0.1, 0.05
Spot-level contrastive learning	Feature masking rate	p_{mask}	0.2, 0.1
Spot-level contrastive learning	Temperature	τ	0.4
Cluster-level contrastive learning	Number of prototypes	K	2
Loss function	Reconstruction weights	λ_1, λ_2	1, 10
Loss function	VGAE weights	λ_3, λ_4	1, 1
Loss function	Contrastive weights	λ_5, λ_6	0.01, 1

Supplementary Table S4. Embedding contribution analysis of the cross-modal masked reconstruction strategy. Linear CKA was used to quantify the similarity between the VGAE fusion output and modality-specific representations. The image/gene ratio was calculated as Sim(Z_{vgae} , Z_{img}) / Sim(Z_{vgae} , Z_{gene}). A higher image/gene ratio indicates a stronger relative contribution of image-derived representations to the VGAE fusion output compared with gene-derived representations. SpatialModal w/o cross-modal masked reconstruction denotes the model variant in which the cross-modal masked reconstruction mechanism was removed.

**Sample**	**Variant**	**Sim(Z_{vgae}, Z_{gene})**	**Sim(Z_{vgae}, Z_{img})**	**Image/Gene ratio**
151509	SpatialModal	0.592	0.517	0.874
	w/o cross-modal masked reconstruction	0.915	0.213	0.233
151672	SpatialModal	0.572	0.477	0.834
	w/o cross-modal masked reconstruction	0.835	0.405	0.485
151674	SpatialModal	0.562	0.425	0.756
	w/o cross-modal masked reconstruction	0.799	0.305	0.381
Mean	SpatialModal	0.575	0.473	0.821
	w/o cross-modal masked reconstruction	0.85	0.308	0.366

Supplementary Table S5. Ablation analysis of cross-modal replacement and mask reconstruction loss. ARI values are reported for three representative DLPFC sections. Full SpatialModal denotes the original model containing both cross-modal replacement and mask reconstruction loss. SpatialModal w/o cross-modal replacement removes the replacement of gene-derived features with matched image-derived features. SpatialModal w/o mask reconstruction loss retains cross-modal replacement but removes the reconstruction constraint for perturbed spots. SpatialModal with random feature masking sets selected gene-derived features to zero instead of replacing them with matched image-derived features. All other model components, training settings, and downstream clustering procedures were kept unchanged.

Sample	SpatialModal	w/o cross-modal replacement	w/o mask reconstruction loss	with random feature masking
151509	0.642	0.404	0.358	0.404
151672	0.724	0.439	0.295	0.433
151674	0.573	0.361	0.433	0.448
Mean	0.646	0.401	0.362	0.428

Supplementary References

1 Long, Y. *et al.* Spatially informed clustering, integration, and deconvolution of spatial transcriptomics with GraphST. *Nat Commun* **14**, 1155 (2023). https://doi.org/10.1038/s41467-023-36796-3

2 Zhang, L., Liang, S. & Wan, L. A multi-view graph contrastive learning framework for deciphering spatially resolved transcriptomics data. *Brief Bioinform* **25** (2024). https://doi.org/10.1093/bib/bbae255

3 Yu, Z. *et al.* Accurate Spatial Heterogeneity Dissection and Gene Regulation Interpretation for Spatial Transcriptomics using Dual Graph Contrastive Learning. *Adv Sci (Weinh)* **12**, e2410081 (2025). https://doi.org/10.1002/advs.202410081

4 Xu, H. *et al.* Unsupervised spatially embedded deep representation of spatial transcriptomics. *Genome Med* **16**, 12 (2024). https://doi.org/10.1186/s13073-024-01283-x

5 Xu, C. *et al.* DeepST: identifying spatial domains in spatial transcriptomics by deep learning. *Nucleic Acids Res* **50**, e131 (2022). https://doi.org/10.1093/nar/gkac901

6 Hu, J. *et al.* SpaGCN: Integrating gene expression, spatial location and histology to identify spatial domains and spatially variable genes by graph convolutional network. *Nat Methods* **18**, 1342–1351 (2021). https://doi.org/10.1038/s41592-021-01255-8

7 Yang, Y. *et al.* STAIG: Spatial transcriptomics analysis via image-aided graph contrastive learning for domain exploration and alignment-free integration. *Nat Commun* **16**, 1067 (2025). https://doi.org/10.1038/s41467-025-56276-0

8 Steinley, D. Properties of the Hubert-Arabie adjusted Rand index. *Psychol Methods* **9**, 386–396 (2004). https://doi.org/10.1037/1082-989X.9.3.386

9 Estevez, P. A., Tesmer, M., Perez, C. A. & Zurada, J. M. Normalized mutual information feature selection. *IEEE Trans Neural Netw* **20**, 189–201 (2009). https://doi.org/10.1109/TNN.2008.2005601

10 Shahapure, K. R. & Nicholas, C. in *2020 IEEE 7th International Conference on Data Science and Advanced Analytics (DSAA).* 747–748.

11 Vergani, A. A. & Binaghi, E. in *2018 IEEE International Conference on Fuzzy Systems (FUZZ-IEEE).* 1–8.

12 Healy, J. & McInnes, L. Uniform manifold approximation and projection. *Nature Reviews Methods Primers* **4**, 82 (2024). https://doi.org/10.1038/s43586-024-00363-x

13 Wolf, F. A. *et al.* PAGA: graph abstraction reconciles clustering with trajectory inference through a topology preserving map of single cells. *Genome Biol* **20**, 59 (2019). https://doi.org/10.1186/s13059-019-1663-x

14 Novotny, B. C. *et al.* SpaFlow: a Nextflow pipeline for QC and clustering of MxIF datasets. *Bioinform Adv* **5**, vbaf032 (2025). https://doi.org/10.1093/bioadv/vbaf032

15 Palla, G. *et al.* Squidpy: a scalable framework for spatial omics analysis. *Nat Methods* **19**, 171–178 (2022). https://doi.org/10.1038/s41592-021-01358-2

16 Jin, S. *et al.* Inference and analysis of cell-cell communication using CellChat. **12**, 1088 (2021). https://doi.org/10.1038/s41467-021-21246-9

17 Schiebinger, G. *et al.* Optimal-Transport Analysis of Single-Cell Gene Expression Identifies Developmental Trajectories in Reprogramming. *Cell* **176**, 928–943.e922 (2019). https://doi.org/10.1016/j.cell.2019.01.006

18 Stiles, J. & Jernigan, T. The Basics of Brain Development. *Neuropsychology review* **20**, 327–348 (2010). https://doi.org/10.1007/s11065-010-9148-4

19 Keefe, M. G., Steyert, M. R. & Nowakowski, T. J. Lineage-resolved atlas of the developing human cortex. *Nature* **647**, 194–202 (2025). https://doi.org/10.1038/s41586-025-09033-8

20 Li, S., Gai, K., Dong, K., Zhang, Y. & Zhang, S. High-density generation of spatial transcriptomics with STAGE. *Nucleic Acids* **52**, 4843–4856 (2024). https://doi.org/10.1093/nar/gkae294

21 Maynard, K. R. *et al.* Transcriptome-scale spatial gene expression in the human dorsolateral prefrontal cortex. *Nat Neurosci* **24**, 425–436 (2021). https://doi.org/10.1038/s41593-020-00787-0

22 Chen, S. *et al.* Spatially resolved transcriptomics reveals genes associated with the vulnerability of middle temporal gyrus in Alzheimer's disease. *Acta Neuropathol Commun* **10**, 188 (2022). https://doi.org/10.1186/s40478-022-01494-6

23 Mantri, M. *et al.* Spatiotemporal single-cell RNA sequencing of developing chicken hearts identifies interplay between cellular differentiation and morphogenesis. *Nat Commun* **12**, 1771 (2021). https://doi.org/10.1038/s41467-021-21892-z

24 Codeluppi, S. *et al.* Spatial organization of the somatosensory cortex revealed by osmFISH. *Nat Methods* **15**, 932–935 (2018). https://doi.org/10.1038/s41592-018-0175-z

25 Chen, K. H., Boettiger, A. N., Moffitt, J. R., Wang, S. & Zhuang, X. RNA imaging. Spatially resolved, highly multiplexed RNA profiling in single cells. *Science* **348**, aaa6090 (2015). https://doi.org/10.1126/science.aaa6090

26 Wang, X. *et al.* Three-dimensional intact-tissue sequencing of single-cell transcriptional states. *Science* **361**, eaat5691 (2018). https://doi.org/10.1126/science.aat5691

27 Chen, A. *et al.* Spatiotemporal transcriptomic atlas of mouse organogenesis using DNA nanoball-patterned arrays. *Cell* **185**, 1777–1792 e1721 (2022). https://doi.org/10.1016/j.cell.2022.04.003

28 Kornblith, S., Norouzi, M., Lee, H. & Hinton, G. in Proceedings of the 36th International Conference on Machine Learning Vol. 97 (eds Chaudhuri Kamalika & Salakhutdinov Ruslan) 3519—3529 (PMLR, Proceedings of Machine Learning Research, 2019).
